# Evolutionary History of Lagomorphs in Response to Global Environmental Change

**DOI:** 10.1371/journal.pone.0059668

**Published:** 2013-04-03

**Authors:** Deyan Ge, Zhixin Wen, Lin Xia, Zhaoqun Zhang, Margarita Erbajeva, Chengming Huang, Qisen Yang

**Affiliations:** 1 Key Laboratory of Zoological Systematics and Evolution, Institute of Zoology, Chinese Academy of Sciences, Beijing, China; 2 Institute of Vertebrate Paleontology and Paleoanthropology, Chinese Academy of Sciences, Beijing, China; 3 Geological Institute, Siberian Branch, Russian Academy of Sciences, Ulan-Ude, Russia; Monash University, Australia

## Abstract

Although species within Lagomorpha are derived from a common ancestor, the distribution range and body size of its two extant groups, ochotonids and leporids, are quite differentiated. It is unclear what has driven their disparate evolutionary history. In this study, we compile and update all fossil records of Lagomorpha for the first time, to trace the evolutionary processes and infer their evolutionary history using mitochondrial genes, body length and distribution of extant species. We also compare the forage selection of extant species, which offers an insight into their future prospects. The earliest lagomorphs originated in Asia and later diversified in different continents. Within ochotonids, more than 20 genera occupied the period from the early Miocene to middle Miocene, whereas most of them became extinct during the transition from the Miocene to Pliocene. The peak diversity of the leporids occurred during the Miocene to Pliocene transition, while their diversity dramatically decreased in the late Quaternary. Mantel tests identified a positive correlation between body length and phylogenetic distance of lagomorphs. The body length of extant ochotonids shows a normal distribution, while the body length of extant leporids displays a non-normal pattern. We also find that the forage selection of extant pikas features a strong preference for C_3_ plants, while for the diet of leporids, more than 16% of plant species are identified as C_4_ (31% species are from Poaceae). The ability of several leporid species to consume C_4_ plants is likely to result in their size increase and range expansion, most notably in *Lepus*. Expansion of C_4_ plants in the late Miocene, the so-called ‘nature’s green revolution’, induced by global environmental change, is suggested to be one of the major ‘ecological opportunities’, which probably drove large-scale extinction and range contraction of ochotonids, but inversely promoted diversification and range expansion of leporids.

## Introduction

Within Lagomorpha, there are two extant families, Ochotonidae (pikas) and Leporidae (hares and rabbits) [1]–[4]. Ochotonids include a single extant genus with 28 species [1], [5]. Their current distribution is confined to plateau-steppe and talus habitats in Asia and North America. Several wild populations are suffering contraction and extirpation [6]–[11]. However, the condition of leporids is quite different from that of ochotonids, the former with 62 extant species (comprising 12 genera) widely distributed in the tropical forest, temperate steppe, plateau, desert and even Arctic areas of Eurasia, Africa, North America and Central America. Several species have been selected as domestic animals. They have been successfully introduced into Australia and the southern part of South America [12]. Certain species have even established stable wild populations in new habitats. For example, *Sylvilagus floridanus* in Northern Italy [13], [14], *Lepus europaeus* in New Zealand [15] and *Oryctolagus cuniculus* in Australia [16], [17]. Despite the wide expansion of these species, several endemic taxa are threatened to some degree, including *Pronolagus*, *Bunolagus*, *Romerolagus*, *Nesolagus*, *Pentalagus*, *Caprolagus*, and some species of *Sylvilagus* and *Lepus* [1]. Fossil records from Western India indicated an Asia origin of lagomorphs, which was dated to the early Eocene [18]. The sister relationship of ochotonids and leporids is widely accepted; however, it is unclear how their distribution ranges differentiated during evolution, and what drove their disparate evolutionary process.

Despite diversification from a common ancestor, the size of the leporids is generally differentiated to that of ochotonids. Body size is one of the most important phenotypic characters that shape the physiological properties of animals, and is closely related to their life history traits and behavior [19]–[21]. It is generally accepted that the maximum size of mammals has increased during the past 65 million years, most prominently in cetaceans [22], [23]. A minimum of 10 million generations has been proposed for terrestrial mammal mass to increase 5,000 fold, with fluctuation in different taxa [22]. However, size increase is not universal, nor at equal rates across the mammals. For example, the mass of the biggest leporids is about 30–40 times that of the biggest ochotonids, while the mass of smallest leporids is slightly larger than the biggest ochotonids. The evolutionary history of large-sized herbivores, taking the savanna-adapted ungulates as an example, show a strong concordance with the origin and expansion of grasslands, and they are well adapted to abrasive diets and fast running [24]. The forage strategies of African Bovidae have been reviewed, with a positive correlation identified between body mass increase and the consumption of monocots [25]. This hypothesis being further supported by stable isotope analysis, which found a correlation of C_4_ biomass proportion and body mass [26]. It is uncertain whether there is a concordance between forage selection and the change of body sizes within Lagomorpha.

As primary consumers in the terrestrial ecosystem and having an abundant fossil record, the Lagomorpha represent ideal models for studying biochronology as well as the evolution of herbivores and their response to global environmental change [27], [28]. However, the focus of previous studies in Paleobiology was mainly in relation to faunal succession in terrestrial deposits, taxonomic description and revision, and morphological diversification [29]–[32]. Meng and McKenna [33] reported that the mammalian faunal composition in northern China and Mongolia changed from perissodactyl-dominant faunas to rodent-lagomorph-dominant faunas during the Eocene/Oligocene transition in Asia, while a thorough study of the evolutionary history of ochotonids and leporids using a comprehensive sample of fossil data has not been carried out since 1967 [34]. The dynamics of lagomorphs in relation to global environmental change has not been fully studied. As for the studies of extant species, the main focus was related to molecular phylogeny, historical biogeography, intraspecific differentiation and population genetics of ochotonids or leporids [2], [35]–[40], whereas a combined study of these animals is lacking. Combining fossil records and molecular data is important for a comprehensive understanding of the evolutionary history of different vertebrates [41]–[43]. For these reasons we consider it timely to compare fossil records between ochotonids and leporids and to reconstruct the evolutionary history of Lagomorpha in the context of global environmental change.

Paleobiological studies suggest that global-scale biodiversity is driven largely by abiotic factors such as climate, landscape and food supply [44]. Our previous study demonstrated a possible relationship between expansion of C_4_ biomass in the late Miocene and wide extinction and range contraction in ochotonids [45]. We hypothesize that the global environmental change, specifically the increase of C_4_ biomass during the transition between Miocene to Pliocene, also influenced the evolutionary trajectory of leporids. In the present study, we aim to compare the evolutionary history of ochotonids and leporids and identify the main events that may have driven their diversification shifts, the differentiation of their distribution ranges and body sizes, as well as gain insights into their future prospects using dietary strategies of extant species. The methods used here mainly following Ge et al. [45].

## Materials and Methods

### 1 Fossil Records

We obtained the bulk of the records from the Paleobiology database (Available: http://paleodb.org/cgi-bin/bridge.pl, Accessed 2012 Dec 1), the Neocene of Old World Database of fossil mammals (Available: http://www.helsinki.fi/science/now/, Accessed 2012 Dec 1), the Miocene Mammal Mapping Project of West United States (Available: http://www.ucmp.berkeley.edu/miomap/, Accessed 2012 Dec 1) and the National Infrastructure of Mineral Rock and Fossil Resources for Science and Technology of China (Available: http://www.nimrf.net.cn/, Accessed 2012 Dec 1). Then we searched the Zoological Records from 1864 to 2012 (Available: http://apps.webofknowledge.com/, Accessed 2012 Dec 1) in addition to the related literature of each genus. To avoid missing information, we checked the fossil occurrences in different epochs: the Latin names of each genus together with the names of different epochs were used as keywords in searches. Several monographs or dissertations summarized the fossil occurrences of Lagomorpha in Asia, Europe, North America and Africa [3], [28], [46]–[49]. This information was also checked to update the fossil records of these animals. The fossil records of ochotonids were updated from Ge et al. [45]. A database including worldwide fossil records of Lagomorpha was established. Taxonomy, localities and epochs were double checked and updated. This database is provided as Table S1.

Geographic coordinates were obtained from the original databases, the original records in the literature or with Google Earth (Available: http://www.google.com/earth/index.html, Accessed 2012 Dec 1). These records were illustrated on the world map by three layers: the first layer included fossil records from the Eocene and Oligocene epochs, the second layer included fossils from the Miocene, and the third layer included fossils from the Pliocene to the recently extinct populations. Arcview version 3.2 was used to visualize the distribution of these fossils. In paleobiology, genera are generally considered better than species for reconstructing evolutionary history of fossil organisms [50], and the generic level diversity has been used frequently in large scale paleoecological analyses [51]–[54]. Here, we compared the occurrences of genera within ochotonids and leporids. The number of genera was counted based on the database updated in the present study.

### 2 Reconstructing Phylogeny and Calibrating Divergence Time

The molecular phylogeny and divergence times within Lagomorpha were reconstructed from three mitochondrial genes, *cytb*, ND4 and 12S. These data were obtained mainly from previous studies of our research group [38], [55] and the data published by Matthee et al. [2]. Accession numbers for *Cytb*, ND_4_ and 12S are given in Table S2. This matrix was generated to include most extant species within Lagomorpha and to avoid large imbalance regarding the lengths of these sequences. *Cytb* was present in each taxon. The sequences were aligned by Clustalw2 [56]. Systematics attribution of these species mainly follows Hoffman and Smith [1].

Four representative genera of Rodentia, together with Primates (2 genera), Scandentia (one genus) and Carnivora (one genus) were used as outgroup taxa (Table S2). 50.2 million years for the divergence of ochotonids and leporids, 69 million years for the crown age of Rodentia and 12 million years for the split of *Mus* and *Rattus* were used as time priors [45], [57]. A relaxed molecular clock analysis was implemented in the program BEAST [58]. The GTR substitution model was used with the dataset being partitioned according to gene and codon position (the protein coding *Cytb* and ND_4_), and their substitution models were unlinked. The Yule speciation prior was used. Two MCMC chains were run for 1000 million generations and sampled every 1000 generations. The first 25% trees of each run were discarded as the burnin phase. The results were examined in Tracer 1.5.0 [59] to confirm the effective sample size for each parameter exceeded 200. We used TreeAnnotator v1.6.1 (Available: http://beast.bio.ed.ac.uk/TreeAnnotator, Accessed 2012 Dec 1) and Figtree v 1.2.2 (Available: http://tree.bio.ed.ac.uk/software/figtree/, Accessed 2012 Dec 1) to annotate and illustrate the final tree.

### 3 Inferring Historical Biogeography

The distributions of terminal taxa included in the above analysis were split into Asia (A), Europe (B), North America (C), South America (D) and Africa (E). Fossil occurrences of extant species were also included in the analyses, for example fossils of *O*. *pussila* are widely distributed in Europe [28], [60]–[63]. Widespread species were coded as present in multiple regions. Inference of ancestral distributions was implemented in S-DIVA version 2 (RASP) [64], [65]. Trees obtained from the Bayesian MCMC analysis were used so as to account for phylogenetic uncertainty. Statistic dispersal-variate analysis (S-DIVA), Bayesian MCMC analysis (Bayes DIVA) and maximum parsimony analysis (MP) [64]–[67] were conducted to test the accuracy and stability of the results.

### 4 Inferring Body Size Evolution

Body lengths of most extant species were obtained from PanTHERIA [68]. However, body length of several species, especially these endemic to China, were missing in this database. Therefore, we obtained permission to check the specimens preserved in the following museums: The Institute of Zoology, Chinese Academy of Sciences (IOZCAS), Kunming Natural History Museum of Zoology (KNHMZ), Northwest Institute of Plateau Biology, Chinese Academy of Sciences (NIPBCAS), and Zoological Institute, Saint-Petersburg, Russia Academy of Sciences (ZISPRAS). We calculated the average body length of these species based on the collection records. Previous studies have demonstrated that sexual dimorphism in Lagomorpha is insignificant [69]–[71], thus the body lengths of adult males and females were combined to calculate the average body length of these species.

The genetic distance among species was calculated from the patristic distances given in the majority consensus tree from the Bayesian inferences. Body size distances among species were exported from the database which considers the average body length of these species as continuous characters. A Mantel test was used to test the correlation between phylogenetic distances and body size distances in NTSYSpc 2.21 [72]. The decision as to whether reconstruction of body lengths of ancestors using extant species was appropriate, was based on this analysis. The parsimony method was used to reconstruct ancestral states of body lengths. The body lengths of ancestors were mapped on the tree reconstructed from above inferences. These analyses were performed using the program Mesquite v2.74 [73]. In order to identify the differences in body size-species richness patterns between ochotonids and leporids, we conducted a Kolmogorov-Smirnov test (KS) and Shapiro-Wilk (SW) test to check for normality of their body length. Body lengths were *log_10_* transformed and a length-frequency distribution was generated for extant ochotonids and leporids.

### 5 Identifying Forage Selection

The likely food plants of extant leporids were compiled from 28 sources (detailed information of these references is listed in Table S3 with References S1–28 in File S1). We compared the family level status of these plants with that of extant pikas [45]. The photosynthetic pathway of these plants was also categorized based on the reported C_4_ plants [74]–[77] and the information provided by the database: The Grass Genera of the World (Available: http://delta-intkey.com/grass/, Accessed 2012 Dec 1). A nonparametric Chi-Square test was performed to compare the prevalence of C_3_ and C_4_ plants in the diets of ochotonids and leporids. The null hypothesis was ochotonids and leporids have equal preference on C_3_ and C_4_ species. There are several sources that have reported the proportion of these plants in the diet of different lagomorphs [References S29–37 in File S1]. We summed the total proportion of C_3_, C_4_ and other compositions (including C_3_–C_4_ intermediates, CAM species and unidentified species) in the summer, wet season or annual diet of these species and illustrated this information on the world map.

## Results

### 1 Evolutionary History of Lagomorpha

Based on fossil records, the stem Lagomorpha were mainly derived in Asia, in China and Mongolia [78]–[82]. Fossil records showed that ochotonids have 32 genera with approximately 180 species formally described, about 150 of which suffered the fate of extinction (updated from [45]). The formally nominated leporids include about 45 genera with more than 190 species, at least 130 of which became extinct. The validity of several species is controversial since the fossil taxa may be oversplit [82], while the occurrences (in both period and locality) provide important information for understanding the long-term evolutionary history of these animals.

**Figure 1:**
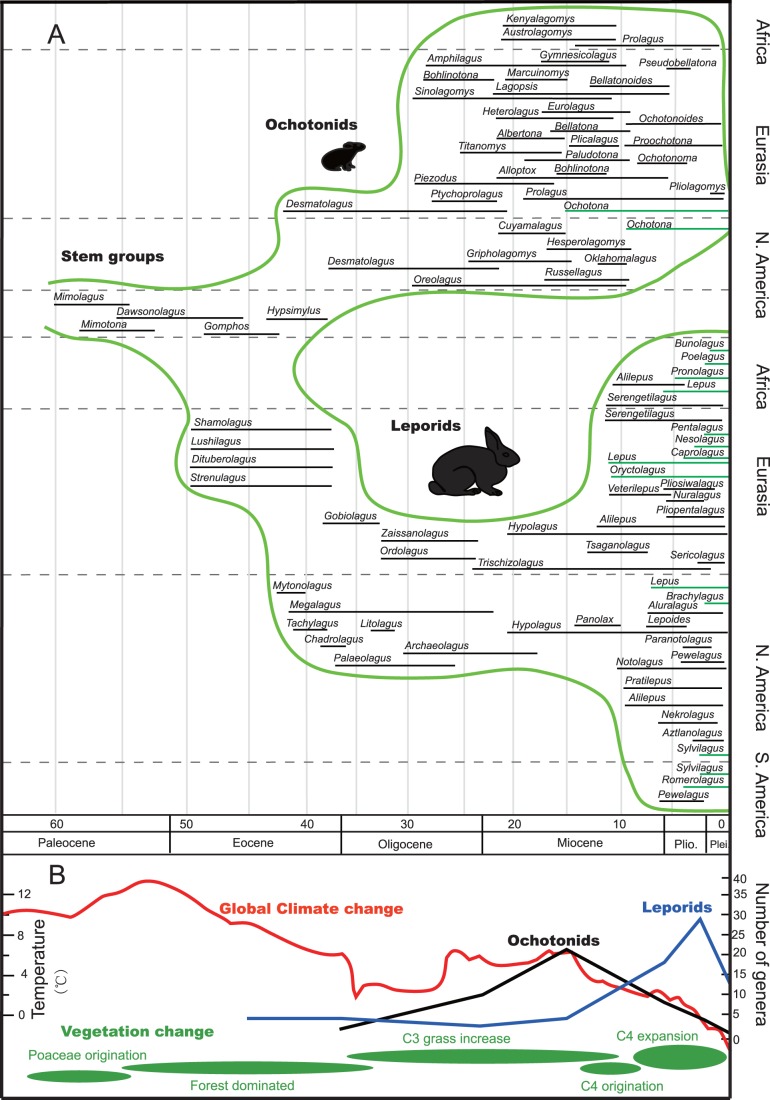
Fossil occurrences of leporids and ochotonids shown alongside global environmental change. A. Occurrences of genera in different epochs. The occurrences of ochotonids are updated from Ge et al. [45]. Black bars give the extinct genera, green bars give the extant genera. The lengths of these bars are based on the maximum age and minimum age of each genus. B. The global climate change (Figure 2 in [110]) and vegetation change [100], [101] and the genera number of ochotonids and leporids.

The earliest ochotonid, *Desmatolagus*, was reported from Eurasia and North America [83]–[87]. The diversification of ochotonids was not substantial during the late Eocene to the Oligocene, while from the late Oligocene to the early Miocene, the speciation of ochotonids accelerated dramatically. Its diversity peaked in the Middle Miocene, with range expansion into South Africa during the early Miocene (Figure 1). However, in the late Miocene, a large number of genera became extinct, with only 3–4 genera surviving the transition from the Miocene to Pliocene. The only extant genus, *Ochotona*, originated in the late Miocene. It is currently distributed in plateau-steppe and talus habitats of Asia and North America.

**Figure 2:**
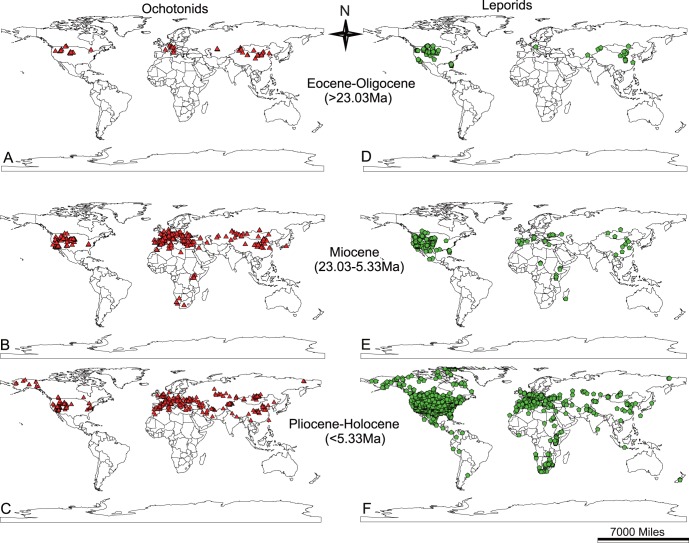
Fossil occurrences of ochotonids and leporids. A, D, Eocene to Oligocene; B, E: Miocene; C, F: Pliocene to Holocene. Triangles show the occurrences of ochotonids, pentagons show the occurrence of leporids. Scale of 7000 miles is at the Equator.

The earliest leporids include the Eurasian *Shamolagus*, *Lushilagus*, *Dituberolagus*, *Strenulagus* etc. and the North American *Mytonolagus*, *Megalagus*, *Tachylagus* etc. (Figure 1). During the middle and late Eocene, there were about 10 genera present in the northern hemisphere, while during the transition from the Eocene to Oligocene, the generic diversity of leporids remained modest. The generic diversity of leporids reached the lowest point during the transition from the Oligocene to the Miocene, with a minor increase in the middle Miocene (Figure 1, 2). The diversification of leporids accelerated from the late Miocene to Pliocene. It thrived during the Pliocene and Pleistocene, with pervasive expansion to Africa and South America (Figure 1, 2). The number of leporid genera substantially decreased in the Holocene. Detailed information of these genera (excluding synonyms), the occurrences of each species together with large numbers of innominated records are listed in Table S1.

### 2 Divergence Time and Distribution Patterns of Lagomorpha

**Figure 3:**
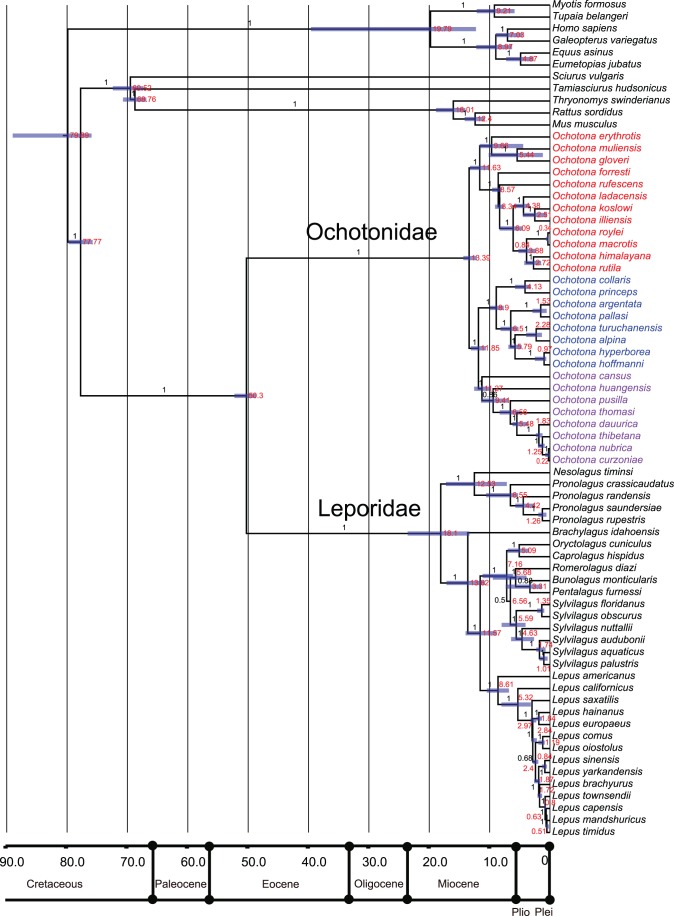
Phylogenetic relationships and divergence times of Lagomorpha. Branch labels on the tree give posterior probabilities. Node labels give median value of divergence time. Blue bars give 95% interval confidence of divergence time. Three ecotype groups of *Ochotona* are marked in different colors: red, the Mountain group; blue, the Northern group; pink, the shrub-steppe group.

**Figure 4:**
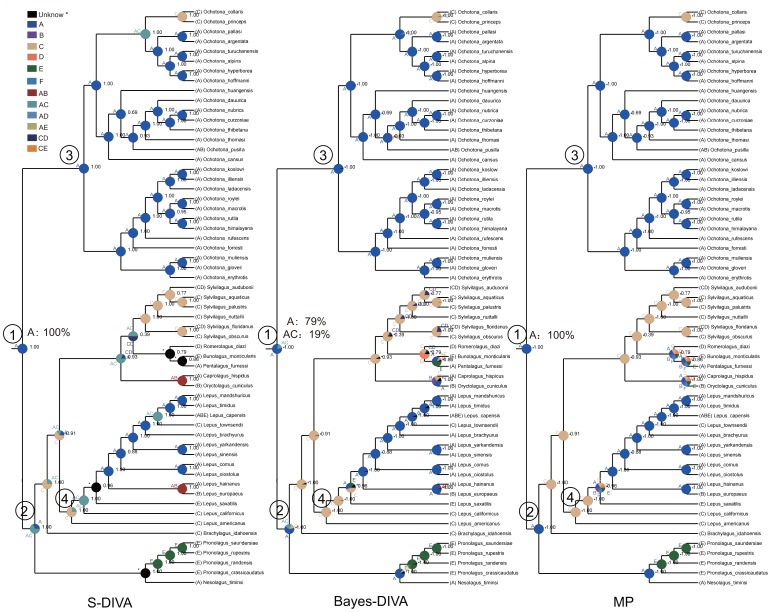
The historical distribution of Lagomorpha inferred from different algorithms: S-DIVA, Bayes-DIVA, Maximum parsimony. The phylogeny was based on the majority consensus trees derived from Bayesian inference analysis of three genes. Posterior probabilities are shown beside each node. Biogeographical regions used in the analysis including: Asia (A), Europe (B), North America (C), South America (D) and Africa (E). Out groups were excluded in the figures. Three methods identified Asia as the origin center of Lagomorpha (Node 1, S-DIVA 100%, Bayes-DIVA 79%, Maximum parsimony, 100%). Asia was also identified as the most probable ancestral region of leporids and ochotonids respectively (Node 2 and 3), while, *Lepus* firstly appeared in North America (Node 4).

**Figure 5:**
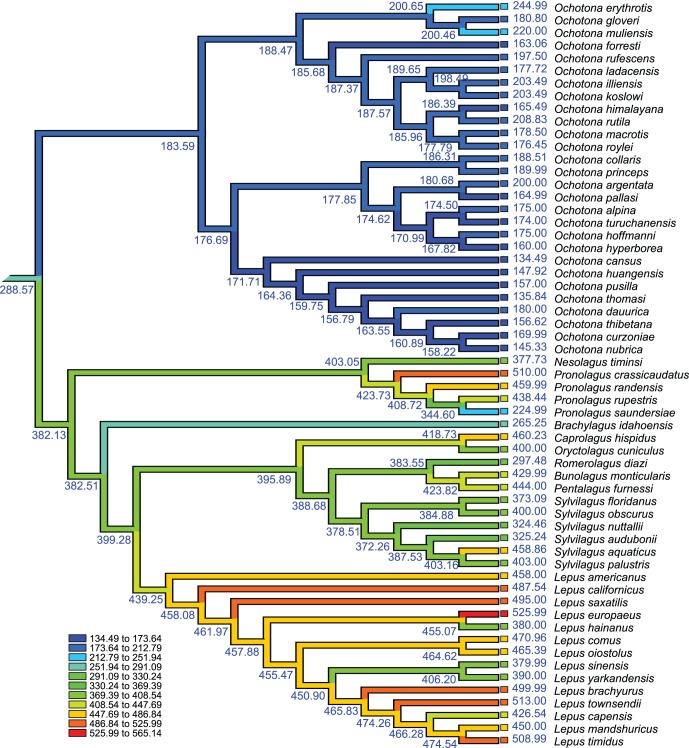
Body size evolution in Lagomorpha. The phylogenetic structure was based on Bayesian inferences. The body lengths of terminal taxa were mainly from PanTHERIA [68] or calculated basing on museum collections. Body length of each node was inferred by parsimony methods. Colors on the branches show the change of body size. The units of body length are millimeters.

Reconstructing the phylogeny of Lagomorpha based on the combined matrix of three genes revealed that the previously recognized three ecotype groups of pika (the shrub-steppe group, the Northern group and the Mountain group) were highly supported (Figure 3) [36], [45], [88]. The phylogenetic structure inferred here was slightly different from the findings of Matthee et al. [2], the latter of which was reconstructed from a combined supermatrix of seven genes (five nuclear and two mitochondria genes). However, these genes were aligned manually in their study. Based on Bayesian inferences of molecular data, the divergence of extant leporids was dated at around 18.1 million years, near the early Miocene. The earliest derived extant genera were *Nesolagus* and *Brachylagus*. *Oryctolagus*, *Caprolagus*, *Romerolagus*, *Bunolagus*, *Pentalagus*, and *Silvilagus* formed a monophyletic group, which split around 7.16 million years. Diversification within *Silvilagus* began around 5.59 million years, and the split of *Lepus* began around the divergence of *Silvilagus* (8.61 million years). *Lepus* from Eurasia diversified primarily in the Pleistocene (<2.5 million years). The extant ochotonids diverged in North America around 8.9 million years, the extant leporids (*Lepus*) expanded from North America to Eurasia and Africa, and diverged around 5.32 million years (Figure 3). Diversification within three ecotypes of ochotonids was similar to the results obtained based on a smaller dataset, focusing on ochotonid taxa [45]. The divergence times of these three groups were 11.63, 8.9 and 11.27 million years. The earliest divergence of African leporids was dated to the late middle Miocene (around 12.5 million years), overlapping slightly with the occurrence of ochotonids in Africa. Based on the distribution of extant species, Asia was identified as the ancestral region of Lagomorpha (S-DIVA, 100%, Bayes-DIVA 79%, MP 100%) (Figure 4, Node 1) and it was also identified as the most probable ancestral region of leporids and ochotonids (Figure 4, Node 2 and Node 3). Meanwhile, the most widely distributed genus of Lagomorpha, *Lepus*, first appeared in North America (Figure 5, Node 4).

### 3 Body Size Evolution of Lagomorpha

**Figure 6:**
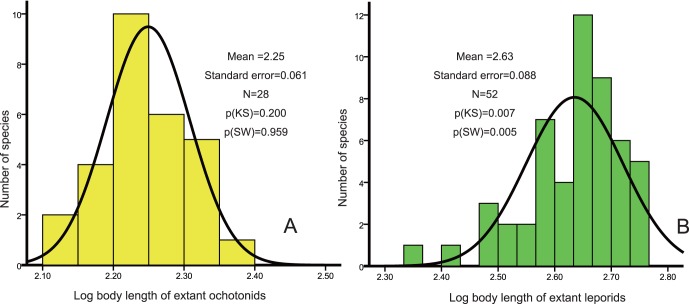
Frequency of log body length for extant ochotonids and leporids. A. Ochotonids, this figure shows a slightly right skewed distribution, with a mode tending to smaller species. B. Leporids, this figure shows a left skewed distribution, with a mode tending to larger species.

Mantel tests indicated that there was a significant correlation between body length variation and phylogenetic distance among different lagomorphs (r = 0.8008, t = 33.7616, P<0.001), permitting the reconstruction of body length for their ancestors. The results showed that ochotonids had a conservative pattern in body length evolution, while the length of leporids was generally greater and more variable, most prominently in *Lepus*. The body length of the most recent common ancestor of Lagomorpha is about 289 mm (Figure 5). For ochotonids, the frequency distribution of *log* body length was normally distributed [Kolmogorov-Smirnov test, p(KS) = 0.200, Shapiro-Wilk test, p(SW) = 0.959], while the *log* body length of leporids displayed a non-normal distribution and prominently left skewed [Kolmogorov-Smirnov test p(KS) = 0.007, Shapiro-Wilk test, p(SW) = 0.005]. Ochotonids showed a mode slightly tending to smaller species, while leporids showed a very prominent trend toward larger species (Figure 6A, B).

### 4 Forage Selection of Lagomorpha

**Figure 7:**
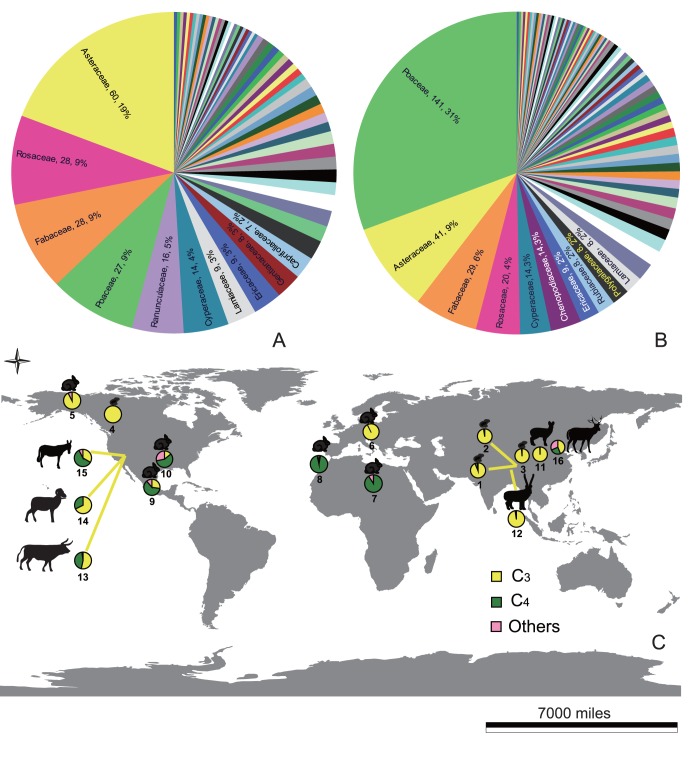
Comparing forage selection of extant laghomorphs and other herbivores. A. Forage selection of extant ochotonids, 63 families were reported as food (consumed directly or collected for hay piles) of extant ochotonids, these plant information mainly follows Ge et al. [45], which was summarized from 29 sources. B. Forage selection of leporids. 91 families were reported as food of leporids. These information was summarized from 28 sources. The top ten families selected by extant ochotonids and leporids are compared with the number of species and their proportions. C. Comparing C_3_, C_4_ and other components in the diet of different herbivores. 1. *Ochotona curzoniae* [Reference S29 in File S1], 2. *Ochotona dauurica* [Reference S29 in File S1], 3. *Ochotona curzoniae* [Reference S30 in File S1]; 4. *Ochotona princeps* [Reference S31 in File S1]; 5. *Lepus timidus* [Reference S32 in File S1]; 6. *Lepus europaeus* [Reference S33 in File S1]; 7. *Lepus flavigularis* [Reference S34 in File S1]; 8. *Oryctolagus cuniculus* [Reference S35 in File S1]; 9. *Sylvilagus floridanus* [Reference S36 in File S1]; 10. *Sylvilagus auduboni* [Reference S37 in File S1]; 11. *Moschus berezovskii* [Reference S38 in File S1]; 12. *Pantholops hodgsoni* [Reference S39 in File S1]; 13. *Bos primigenius* [Reference S40 in File S1]; 14. *Ovis canadensis* [Reference S40 in File S1]; 15. *Equus africanus asinus* [Reference S40 in File S1]; 16. *Elaphurus davidianus* [Reference S41 in File S1].Scale of 7000 miles is at the Equator.

Food plants of ochotonids were summarized from 29 references, with about 322 species identified, 19% of them belonging to Asteraceae. These food plants represent 63 families (details are listed in the supplementary documents of [45]). According to the findings of 28 sources, there were more than 430 species of plants representing nearly 300 genera and 90 families, recorded as the selections of 20 leporid species. These plants were mainly from Poaceae (31%), Astereceae (9%), Fabaceae (6%) and Rosaceae (4%) (Figure 7A, B). Most leporids are generalist herbivores, with only a few species (with limited distributions) specializing on particular plant species. For example, sagebrush (*Artemisia tridentate*) comprises up to 99% of winter and 50% of summer diet of pygmy rabbits (*Brachylagus idahoensis*) [89], [90].

Categorizing the photosynthetic pathway of these plants indicated that the food plants of ochotonids were mainly C_3_, with only 3 species reported as using C_4_ [45]. As for the food plants of leporids, about 16% of species were categorized as C_4_. Preference on C_4_ varies among different genera. For example, the early diverged genera *Nesolagus* and *Brachylagus* depend mainly on C_3_ plants, while the later diverged leporid genera show a preference for C_4_ plants, most notably *Caprolagus*. The most widely distributed genus, *Lepus*, showed about 10% of diet species as C_4_ plants (Figure 7)_._ This percentage is far beyond the proportion of C_4_ terrestrial plant species in nature (3–4%). Particularly, *L. flavigularis* in Mexico, shows a high dependence on C_4_ plants (11 of the 15 species are C_4_). A table including detailed information for forage selection of different leporids is given in Table S3.

Nonparametric Chi-Square test indicated the prevalence of C_3_ and C_4_ plants in the diets of ochotonids and leporids was significantly different (P<0.01, df = 1). The dietary proportions of C_3_, C_4_ and the other plants in the 10 wild populations of Lagomorpha were illustrated on the world map, and demonstrate a high dependence of C_3_ plants in pikas and leporids in areas of high altitude and high altitude. In contrast, *Lepus flavigularis* in the southeastern Oxaca, Mexico, *Oryctolagus cuniculus* in a semiarid Atlantic island (Alegranza, Canarian Archipelago), *Sylvilagus floridanus* at Ixtacuixtla, central Mexico and *Sylvilagus auduboni* in Texas Panhandle Playa basins showed a prominent dependence on C_4_ plants (Figure 7) [References S34–37 in File S1].

## Discussion

### 1 The Evolutionary History of Lagomorpha in Response to Global Environmental Change

The phylogenetic reconstruction based on Mitochondrial DNA or multiple genes generally suggests that placental mammals diverged around the Cretaceous to Paleogene boundary (65.5 million years) [91], [92]. Extinction of non-avian dinosaurs in this period opened the door for the diversification of terrestrial mammals [93]. In the past 65 million years, the natural environment has changed greatly. These changes include the drift and reconnection of continents, the dramatic decline of CO_2_ (in the late Miocene) and the formation of ice-age climate cycles [94]. These events changed the structure and functioning of the terrestrial ecosystem, impacting the floral and faunal composition of different regions. It has been reported that the evolution of large mammals was greatly influenced by these events, particularly the climate-induced expansion of C_4_ biomass in the late Miocene [95], [96]. Based on the data investigated in the present study, the primary consumer, Lagomorpha, was also influenced by these events. Lagomorpha split from stem rodents around the Paleocene-Eocene boundary [18], [97]. The Asian origin of Lagomorpha is widely accepted [18], [29], [97]. In the present study, historical biogeographic inferences based on molecular data is also congruent with these studies. However, two major groups of Lagomorpha, ochotonids and leporids, responded to global environmental changes differently.

Ochotonids originated in Asia during the period between the late Eocene and the early Oligocene, thriving in Eurasia and North America from the late Oligocene to middle Miocene (a period of “global climate optimum”) (Figure 1, 2). They even dispersed to Africa in the early and middle Miocene (Figure 1, 2), when a higher CO_2_ level resulted in a warmer temperature, conditions corresponding to a continent densely covered with forests [98], [99] and increased distribution of C_3_ grasses [100], [101] (Figure 1). The most frequently selected plant families of extant pikas (Figure 6), Asteraceae, Rosaceae and Fabaceae contain large number of C_3_ grasses. Asteraceae experienced a rapid radiation during the Oligocene to middle Miocene. A large number of genera originated within this period [102]–[104].

The origination of leporids in the Eocene occurred within a warmer and wetter climate (Figure 1) [99]. With a trend of global cooling during the transition of Eocene to Oligocene, fossil occurrences of leporids increased slowly. Even in the global climate optimum of the middle Miocene, the diversity of leporids was still unremarkable, with only two sparse genera present in Eurasia and North America, and very few occurrences in Africa (indeterminate species or genera). Plant fossils from the early to middle Miocene indicate that forests and swamps dominated the vegetation in the northern hemisphere [105], [106]. The low diversity of leporids in the early to middle Miocene may indicate that the vegetation of the time did not promote diversification and dispersal of these animals.

In the late Miocene, a period of global cool and dry conditions arrived and as a consequence, the diversity of ochotonids and leporids largely differentiated. Ochotonids disappeared from Africa and continued to develop in both Eurasia and North America (Figure 1, 2), but the number of genera in each continent dramatically decreased (Figure 1). However, the leporids prospered greatly after the late Miocene. A large number of genera arose during the late Miocene to Pliocene period, and some even expanded to Africa and South America (Figure 1). The evolutionary history of leporids is similar to several taxa within Bovidae and Equidae, which showed a high diversity in the period of transition between Miocene and Pliocene [107].

The differing fate of ochotonids and leporids in the late Miocene occurred during the same environmental conditions: a global cool and dry period. This was accompanied by seasonal or regionally imbalanced rainfall in the terrestrial ecosystem, and the depletion of CO_2_ in the atmosphere [108]–[110]. The uplift of the Qinghai Tibet Plateau and the formation of Asian monsoon accelerated the aridity in the North Hemisphere [111]. During the ‘Global green revolution‘ in the late Miocene [112], the predominance of C_3_ plants in tropical and temperate areas was replaced with that of C_4_ plants [95], [113]–[115]. The dominance of the ancestral photosynthetic pathway (C_3_) was challenged by the C_4_ pathway, which evolved independently in more than 45 plant families [75], [76], [116], particularly in the Poaceae. It has been reported that the diversification of C_4_ plants within Poaceae accelerated from the late Miocene to Pliocene, and large number of species in several subfamilies originated during this period [117]. In the following period, a large area of forest on the earth was replaced by open grassland, in the ‘Nature’s green revolution’ [112]. This event probably first started in North America [112], [118], where a large number of open grassland dwellers likely originated. Ochotonids suffered from habitat loss and fragmentation in the temperate and tropical area of Africa, Eurasia and North America [45], while leporids benefited from the replacement of forest by more open C_4_ grassland in the terrestrial ecosystem.

Based on the historical biogeography of Lagomorpha inferred in the present study, we deduce that ancient taxa of these animals possibly lived in the forests of Asia, since the earliest fossils were unearthed from coal mines in Western India [18]. The earliest grassland dwellers within Lagomorpha possibly appeared in North America, where the grassland ecosystem originated much earlier than in other continents [118]. The Bering land bridge served as an important corridor between Eurasia and North America. The two continents became connected by land bridging in the middle Cretaceous and remain joined occasionally from the Eocene until the end of the late Miocene [119]. This connection appeared occasionally during the glacial periods, permitting the dispersal of the ochotonids and leporids between these two continents. The earliest leporid genera occupying these two continents were similar in morphology [120]. The gradual closure of the Turgai Straits advanced the dispersal of both ochotonids and leporids in Asia and Europe, as demonstrated by several taxa widely distributed in those two regions. Extensive exchanges of terrestrial animals between Eurasia and Africa were thought to be initiated by the formation of the ’Gomphotherium Landbridge‘ during the early to middle Miocene. Ochotonids expanded to Africa during this period [46], but became extinct in Africa during the late Miocene. However, the earliest arrival of leporids in Africa is still controversial [46].

It is worth noting that a large number of leporids became extinct after the Pleistocene (Figure 1). This extinction is likely correlated with the extreme climate conditions during the Quaternary glacial periods and a human population which increased during the past 50, 000 years. These two events were thought to have left a measurable negative footprint on biodiversity [121].

### 2 Body Size Evolution of Lagomorpha

The body size of animals is considered to be determined mainly by evolutionary history and ecological conditions. Here, we identified a strong correlation between body length and phylogenetic distance, which indicates that the body size differentiation of lagomorphs may parallel the phylogenetic diversity of these animals. The early diverging taxa are smaller in size, for example *Ochotona*, *Nesolagus* and *Brachylagus*, while the later diverged species are larger in size (the younger species within *Lepus* for instance). These data provide further evidence for the linear correlation between phylogenetic diversity and functional diversity of mammals [122]. It is commonly believed that the maximum body size of terrestrial mammals and the range of body sizes have increased over evolutionary time [93], [123]–[125]. The body size of ochotonids has evolved slowly, remaining a size similar to that of their ancestors (Figure 5), a pattern also very common in the sister taxon of Lagomorpha, Rodentia. However, the close relatives of ochotonids, leporids, are exceedingly variable in size, with most of them enlarged (Figure 5).

Stable carbon isotope analysis on the teeth of Cenozoic Mammalian herbivores from America indicates that the C_3_ plants were predominant in the diet of these animals until the late Miocene (∼8 Ma) [126], then changing to a mixture of C_3_ and C_4_ or C_4_-dominanted food after the late Miocene [96], [127]. However, several endemic species still maintain a high dependence on C_3_, such as musk deers (*Moschus berezovskii*) and Tibetan antelopes (*Pantholops hodgsoni*) (Figure 7C) [References S38–39 in File S1], species relatively smaller compared to their sister taxa. Nevertheless, cattle (*Bos primigenius*), bighorn (*Ovis Canadensis*), burro (*Equus africanus asinus*) and David’s deer (*Elaphurus davidianus*) [References S40–41 in File S1], are all capable of digesting C_4_ plants (Figure 7C), and are larger in size. Analysis of the forage selection of extant ochotonids and leporids demonstrated that ochotonids maintain a predominantly C_3_ diet [45], while most species within leporids (except the early diverging genera) could also handle C_4_ plants successfully (Table S3). Digesting different kinds of plants is evolutionarily challenging in different herbivores, especially considering that the fermentation of cellulose, hemicelluloses and lignins rich in C_4_ plants requires tailored gut microbe communities, which show a close co-evolutionary history with the host [128]. This evidence suggests the food plants are quite relevant to body size evolution in these animals.

In addition to food preference, body size evolution of mammals is probably also correlated with the natural conditions of their habitats, particularly, the size and environmental condition of home ranges [129]. Quintana [130] reported the occurrence of a giant rabbit species in the Balearic Islands of Spain, the 12 kg *Nuralagus rex,* which was living under the conditions of an environment characterized by absence of predators and low levels of resource supply. This kind of insular giantism appears to be a general evolutionary rule for the otherwise small mammals [131]. However, the unique morphological characters of *Nuralagus rex* also impacted its locomotion and neurological activities, possibly leading to its extinction [130]. As opposed to *Nuralagus rex*, extant species of lagomorph which are confined to islands or highly fragmented habitats tend to remain small in body size. For example, the pygmy rabbit *Brachylagus idahoensis* from the central region of North America, *Lepus yarkandensis* from the Tarim Basin of China and *Lepus hainanus* from the Hainan island of China. However, species living in alpine or near arctic areas (e.g. *Lepus oiostolus and Lepus timidus*) are generally larger than other species. The body size evolution of lagomorphs is much more complex than we have previously assumed, their extent and rate of body size change will have to be investigated with a more comprehensive study of fossils and extant species.

### 3 Forage Preference and the Fate of Lagomorphs

The forage selection and historical biogeography of ochotonids were recently discussed [45]. It was inferred that the expansion of C_4_ probably drove extinction and range contraction of ochotonids, since these species show prominent preference on C_3_ plants. C_4_ plants comprise only 3–4% of the vascular plant species, and now contribute 20–30% percent of terrestrial carbon fixation [118], [132]. They are well adapted to arid areas. At the global scale, increasing variability of seasonal rainfall and overgrazing correspondingly lower the C/N ratio of the grassland, hence accelerating the expansion of C_4_ grasses [133], [134]. Leporids, particularly species within *Lepus,* which can digest C_4_ plants successively, are expanding to wider ranges. The continuing success of these animals is likely promoted by the natural or human induced C_4_ plant expansion, particularly the species within Poaceae [135]. The fate of other C_3_ consumers is similar to ochotonids, for example within the North American Equidae, *Onohippidum*, *Cormohipparion*, *Dinohippus*, *Phiohippus*, and *Nannippus* are extinct, while the only extant equid, *Equus*, is well adapted to C_4_ plants [136], [137]. Two endemic species of China, Musk deer and Tibetan antelopes [References S38–39 in file S1], which both require C_3_ plants (Figure 7C), are also classified as endangered.

Despite a distinct evolutionary history, the food preference probably influences the survival prospectives of both ochotonids and leporids. According to the most up to date version of IUCN, one species within Lagomorpha is newly extinct, five of them are near threatened, five of them are vulnerable, three species are now endangered and in total 31 show a tendency to decreasing in the wild [1], [138]–[139]. However, their decrease appears overlooked (possibly due to their small size), despite them being considered a keystone species in several fragile ecosystems [139], [140]. Under the conditions of global warming, the endemic species within Leporidae (possibly include other endemic herbivorous mammals), confined to a small region and relying on C_3_ plants, will probably face the same kind of challenge to that of ochotonids.

### Conclusions

Although ochotonids and leporids have a common Asian origin, the distribution range and body size of these animals is largely differentiated. The different evolutionary trajectories of these two taxa were possibly driven by the global vegetation change, which was induced by climate change. The thriving of ochotonids in the early to middle Miocene probably was related to the prospering of C_3_ grasses under the ‘climatic optimum’. However, the expansion of C_4_ plants in the late Miocene, which was linked to global cooling, continental aridification as well as monsoon intensification, likely induced large scale extinction and range contraction of ochotonids, but inversely promoted diversification and range expansion of leporids. The extreme environmental conditions during the Quaternary glaciation together with the world-wide increase of human population in the Holocene possibly accelerated large scale extinction within leporids. Herbivorous mammals that show less selectivity in food are more likely confined to a small region and are usually conservative in body size evolution. These animals are also present as early diverged species within different taxa, for example, Pygmy rabbit in Leporidae, Musk deer in Moschidae [141] and Tibetan antelope in Bovidae [142], all show primitive phylogenetic positions. With the continuing global warming, the food availability of C_3_ herbivorous mammals might be threatened more heavily. These species may face a future as bleak as the pikas.

## Supporting Information

Table S1 **The known fossil occurrences of Lagomorpha.** The maximum age and minimum age of these genera were summarized from the Paleobiology database (Available: http://paleodb.org/cgi-bin/bridge.pl, Accessed 2012 Dec 1), the Neocene of Old World Database of fossil mammals (Available: http://www.helsinki.fi/science/now/, Accessed 2012 Dec 1), the Miocene Mammal Mapping Project of West United States (Available: http://www.ucmp.berkeley.edu/miomap/, Accessed 2012 Dec 1). There are different viewpoints regarding the higher-level classification of pikas and leporids, here we use ochotonids and leporids to separate these two groups since the taxonomy is not the focus of our present study.
(XLS)

Table S2 **Genbank accessions of Lagomorpha species.**
(DOC)

Table S3 **Plants selected for food by extant leporids.**
(DOC)

File S1 **Supplementary references.**
(DOC)
