# Supplementary material for: Evolutionary History of Lagomorphs in Response to Global Environmental Change
Source: PLoS One. 2013 Apr 3;8(4):e59668. doi: 10.1371/journal.pone.0059668 (PMC3616043; doi:10.1371/journal.pone.0059668)
Supplement: Table S2 — Genbank accessions of Lagomorpha species. (DOC) [file pone.0059668.s002.doc]

**Table S2.** Genbank accessions of DNA sequences

| Taxon | *Cytb* | ND4 | 12S |
| --- | --- | --- | --- |
| *Brachylagus idahoensis* | AY292721.1 | Missing | U58921.1 |
| *Bunolagus monticularis* | AY292718.1 | Missing | U58922.1 |
| *Caprolagus hispidus* | AY292719.1 | Missing | AY292693.1 |
| *Eumetopias jubatus* | NC_004030.2 | Missing | GU475464.1 |
| *Galeopterus variegates* | NC_004031.1 | Missing | NC_004031.1 |
| *Homo sapiens* | HQ157984.1 | HQ157984.1 | HQ157984.1 |
| *Lepus americanus* | AY292733.1 | Missing | AY292707.1 |
| *Lepus brachyurus* | AB058616.2 | Missing | AB058612.1 |
| *Lepus californicus* | AY292731.1 | Missing | AY292705.1 |
| *Lepus capensis* | NC_015841.1 | NC_015841.1 | NC_015841.1 |
| *Lepus comus* | AJ279408.1 | HM232958.1 | AY745181.1 |
| *Lepus europaeus* | NC_004028.1 | NC_004028.1 | NC_004028.1 |
| *Lepus hainanus* | AY745114.1 | HM232861.1 | AY745184.1 |
| *Lepus mandshuricus* | DQ793162.1 | HM232882.1 | AY745159.1 |
| *Lepus oiostolus* | AY599081.1 | HM232945.1 | AY745177.1 |
| *Lepus saxatilis* | AY292730.1 | Missing | AY292704.1 |
| *Lepus sinensis* | AJ279419.1 | HM232972.1 | AY745173.1 |
| *Lepus timidus* | AJ279424.1 | HM232891.1 | AY745157.1 |
| *Lepus townsendii* | AY292729.1 | Missing | AY292703.1 |
| *Lepus yarkandensis* | AY745123.1 | HM232929.1 | AY745170.1 |
| Myotis formosus | HQ184048.1 | HQ184048.1 | HQ184048.1 |
| *Mus musculus* | AY172335.1 | AY172335.1 | AY172335.1 |
| *Nesolagus timminsi* | JN709879.1 | Missing | HQ596485.1 |
| *Ochotona alpina* | EF567055.1 | AF273130.1 | AF348080.1 |
| *Ochotona argentata* | AF272996 | AF273118 | AY012127.1 |
| *Ochotona cansus* | AF273005.1 | AF273128.1 | AJ537415.1 |
| *Ochotona collaris* | EU549736.1 | AF348080 | Missing |
| *Ochotona curzoniae* | FJ227478.1 | AF273124.1 | Missing |
| *Ochotona dauurica* | EF567059.1 | AF273135.1 | Missing |
| *Ochotona erythrotis* | AF272999.1 | AF273121.1 | Missing |
| *Ochotona forresti* | AF272998.1 | AF273120.1 | Missing |
| *Ochotona gloveri* | AF056602.1 | Missing | Missing |
| *Ochotona himalayana* | AF272997.1 | AF273119.1 | Missing |
| *Ochotona hoffmanni* | HM594687.1 | Missing | Missing |
| *Ochotona huangensis* | AF272995.1 | AF273117.1 | Missing |
| *Ochotona hyperborea* | EF567053.1 | EU549756.1 | Missing |
| *Ochotona koslowi* | AF272993.1 | AF273116.1 | Missing |
| *Ochotona illensis* | AY191824 | Missing | Missing |
| *Ochotona ladacensis* | AF272992.1 | AF273114.1 | Missing |
| *Ochotona macrotis* | AF273010.1 | AF273133.1 | Missing |
| *Ochotona muliensis* | AF421884.1 | Missing | Missing |
| *Ochotona nubrica* | AF272991.1 | AF273113.1 | Missing |
| *Ochotona pallasi* | AF272990 | AF273118.1 | Missing |
| *Ochotona princeps* | AF272989.1 | AF273112.1 | AY292690.1 |
| *Ochotona pusilla* | HM366945.1 | Missing | Missing |
| *Ochotona roylei* | AF272988.1 | AF273131.1 | Missing |
| *Ochotona rufescens* | AJ132206.1 | Missing | Missing |
| *Ochotona rutila* | AF515733.1 | Missing | Missing |
| *Ochotona thibetana* | AF272986.1 | AF273110.1 | Missing |
| *Ochotona thomasi* | AF272987.1 | AF273111.1 | Missing |
| *Ochotona turuchanensis* | EF567056.1 | Missing | Missing |
| *Oryctolagus cuniculus* | NC_001913.1 | NC_001913.1 | NC_001913.1 |
| *Pentalagus furnessi* | AY292720.1 | Missing | AB058614.1 |
| *Pronolagus crassicaudatus* | AY292738 | Missing | U31045.1 |
| *Pronolagus randensis* | AY292737.1 | Missing | U31046.1 |
| *Pronolagus rupestis* | AY292735.1 | Missing | AY292711.1 |
| *Pronolagus saundersiae* | AY292736.1 | Missing | AY292712.1 |
| *Rattus sordidus* | GU570665.1 | GU570665.1 | GU570665.1 |
| *Romerolagus diazi* | AY292734.1 | Missing | GU570665.1 |
| *Sciurus vulgaris* | AJ238588.1 | AJ238588.1 | AJ238588.1 |
| *Sylvilagus aquaticus* | AY292726 | Missing | AY292708.1 |
| *Sylvilagus audubonii* | AY292722.1 | Missing | AJ238588.1 |
| *Sylvilagus floridanus* | AY292724.1 | Missing | AY292700.1 |
| *Sylvilagus nuttallii* | AY292723.1 | Missing | U67285.1 |
| *Sylvilagus obscurus* | AY292725.1 | Missing | U58929.1 |
| *Sylvilagus palustris* | AY292727.1 | Missing | AY292697.1 |
| *Tamiasciurus_hudsonicus* | AF147643.1 | Missing | AY227555.1 |
| *Thryonomys swinderianus* | AJ301644.1 | AJ301644.1 | AJ301644.1 |
| *Tupaia belangeri* | NC_002521.1 | NC_002521.1 | NC_002521.1 |
