# Supplementary material for: Evolutionary History of Lagomorphs in Response to Global Environmental Change
Source: PLoS One. 2013 Apr 3;8(4):e59668. doi: 10.1371/journal.pone.0059668 (PMC3616043; doi:10.1371/journal.pone.0059668)
Supplement: Table S3 — Plants selected for food by extant leporids. (DOC) [file pone.0059668.s003.doc]

**Table S3. Plants selected for food by extant** leporids

| **Plant species** | **Leporids species** | **Plant Family** | **Photosynthesis**  **pathway of plants** | **References** |
| --- | --- | --- | --- | --- |
| *Acacia longifolia* | *Oryctolagus cuniculus* | Fabaceae | C3 | Alves et al., 2006 |
| *Acer japonicum* | *Lepus brachyurus* | Sapindaceae | C3 | Shimizu and Shimano, 2010 |
| *Acer mono var. mayril* | *Lepus brachyurus* | Sapindaceae | C3 | Shimizu and Shimano, 2010 |
| *Acer palmatum var. matsumurae* | *Lepus brachyurus* | Sapindaceae | C3 | Shimizu and Shimano, 2010 |
| *Acer rubrum* | *Sylvilagus floridanus*  *Sylvilagus obscurus* | Sapindaceae | C3 | Chapman et al., 1980  Hartman and Barry, 2010 |
| *Acer rufinerve* | *Lepus brachyurus* | Sapindaceae | C3 | Shimizu and Shimano, 2010 |
| *Achillea millefolium* | *Lepus timidus*  *Brachylagus idahoensis* | Asteraceae | C3 | Green and Flinders, 1980;  Seccombe-Hett and Turkington, 2008 |
| *Aciphylla colensoi* | *Lepus europaeus* | Apiaceae | C3 | Flux, 1967 |
| *Actinidia polygama* | *Lepus brachyurus* | Actinidiaceae | C3 | Shimizu and Shimano, 2010 |
| *Adenostemma lavenia* | *Pentalagus furnessi* | Asteraceae | C3 | Yamada, 2008 |
| *Ageratum corymbosum* | *Sylvilagus floridanus* | Asteraceae | C3 | Hudson et al., 2005 |
| *Agropyron repens* | *Lepus europaeus*  *Sylvilagus floridanus* | Poaceae | C3 | Chapman et al., 1980  Reichlin et al., 2006  Katona et al., 2010 |
| *Agropyron smithii* | *Sylvilagus auduboni* | Poaceae | C3 | Scribner and Krysl, 1982 |
| *Agropyron sp.* | *Brachylagus idahoensis* | Poaceae | C3 | Green and Flinders, 1980 |
| *Agropyron* sp. | *Lepus californicus* | Poaceae | C3 | Maccracken and Hansen, 1984 |
| *Agropyron* sp. | *Sylvilagus nuttalli* | Poaceae | C3 | Maccracken and Hansen, 1984 |
| *Agrostis hesperia* | *Lepus granatensis* | Poaceae | C3 | Pauperio and Alves, 2008 |
| *Agrostis* sp. | *Lepus granatensis* | Poaceae | C3 | Pauperio and Alves, 2008 |
| *Agrostis* sp. | *Oryctolagus cuniculus* | Poaceae | C3 | Alves et al., 2006 |
| *Aizoon canariense* | *Oryctolagus cuniculus* | Aizoaceae | C3 | Martin et al., 2003 |
| *Alchemilla sebaldiaefolia* | *Romerolagus diazi* | Rosaceae | C3 | Fa and Bell, 1990 |
| *Alchemilla sibbaldiifolia* | *Romerolagus diazi* | Rosaceae | C3 | Cervantes and Martinez, 1992 |
| *Alnus arguta* | *Romerolagus diazi* | Betulaceae | C3 | Cervantes and Martinez, 1992 |
| *Alpinia alughah* | *Caprolagus hispidus* | Zingiberaceae | C3 | Bell et al., 1990 |
| *Amaranthus retroflexus* | *Lepus europaeus*  *Sylvilagus auduboni* | Amaranthaceae | C4 | Scribner and Krysl, 1982;  Reichlin et al., 2006 |
| *Ambrosia psilostacha* | *Sylvilagus auduboni* | Asteraceae | C3 | Scribner and Krysl, 1982 |
| *Ambrosia tomentosa* | *Sylvilagus auduboni* | Asteraceae | C3 | Scribner and Krysl, 1982 |
| *Ambrosia trifida* | *Sylvilagus floridanus* | Asteraceae | C3 | Chapman et al., 1980 |
| *Amelanchier* sp. | *Sylvilagus obscurus* | Rosaceae | C3 | Hartman and Barry, 2010 |
| *Ammophila arenaria* | *Oryctolagus cuniculus* | Poaceae | C3 | Alves et al., 2006 |
| *Amorpha fruticosa* | *Lepus brachyurus* | Fabaceae | C3 | Shimizu and Shimano, 2010 |
| *Angiocarpus* sp. | *Lepus granatensis* | Cactaceae | C3 | Pauperio and Alves, 2008 |
| *Anistome filifolia* | *Lepus europaeus* | Apiaceae | C3 | Flux, 1967 |
| *Antennaria rosea* | *Brachylagus idahoensis* | Asteraceae | C3 | Green and Flinders, 1980 |
| *Antennaria* sp. | *Sylvilagus nuttalli* | Asteraceae | C3 | Maccracken and Hansen, 1984 |
| *Anthirrhinum majus* | *Oryctolagus cuniculus* | Plantaginaceae | C3 | Alves et al., 2006 |
| *Anthoxanthum aristatum* | *Lepus granatensis* | Poaceae | C3 | Pauperio and Alves, 2008 |
| *Anthoxanthum odoratum* | *Lepus granatensis* | Poaceae | C3 | Pauperio and Alves, 2008 |
| *Aracea sp. (Homalomena)* | *Nesolagus netscheri* | Araceae | C3 | Flux, 1967 |
| *Aristida divaricata* | *Sylvilagus floridanus* | Poaceae | C4 | Hudson et al., 2005 |
| *Aristida schiedeana* | *Sylvilagus floridanus* | Poaceae | C4 | Hudson et al., 2005 |
| *Aristotelia cockaynei* | *Lepus europaeus* | Elaeocarpaceae | C3 | Flux, 1967 |
| *Arrhenatherum elatius* | *Lepus europaeus* | Poaceae | C3 | Reichlin et al., 2006 |
| *Artemisia tridentata* | *Brachylagus idahoensis*  *Lepus californicus*  *Sylvilagus nuttalli*  *Brachylagus idahoensis* | Asteraceae | C3 | Maccracken and Hansen, 1984  Shipley et al., 2006 |
| *Artemisia tripartite* | *Brachylagus idahoensis* | Asteraceae | C3 | Green and Flinders, 1980 |
| *Artemisia vulgaris* | *Lepus europaeus* | Asteraceae | C3 | Reichlin et al., 2006 |
| *Arunda* sp*.* | *Caprolagus hispidus* | **Poaceae** | C3 | Bell et al., 1990 |
| *Arundinaria gigantea* | *Sylvilagus aquaticus* | Poaceae | C3 | Terrel, 1972 |
| *Aspilia* sp. | *Pronolagus crassicaudatus* | Asteraceae | C3 | Stewart, 1971 |
| *Aster pilosus* | *Sylvilagus aquaticus* | Asteraceae | C3 | Terrel, 1972 |
| *Astragalus* sp. | *Brachylagus idahoensis* | Fabaceae | C3 | Green and Flinders, 1980 |
| *Astragalus* sp. | *Lepus californicus* | Fabaceae | C3 | Maccracken and Hansen, 1984 |
| *Astragalus* sp. | *Sylvilagus nuttalli* | Fabaceae | C3 | Maccracken and Hansen, 1984 |
| *Atriplex* sp. | *Lepus californicus* | Chenopodiaceae | C4 | Maccracken and Hansen, 1984 |
| *Atriplex* sp. | *Sylvilagus nuttalli* | Chenopodiaceae | C4 | Maccracken and Hansen, 1984 |
| *Aucuba japonica var. borealis* | *Lepus brachyurus* | Garryaceae | C3 | Shimizu and Shimano, 2010 |
| *Avena barbata* | *Oryctolagus cuniculus* | Poaceae | C3 | Alves et al., 2006 |
| *Avenella flexuosa* | *Lepus europaeus* | Poaceae | C3 | Reichlin et al., 2006 |
| *Balsamorhiza sagittata* | *Lepus californicus*  *Sylvilagus nuttalli* | Asteraceae | C3 | Maccracken and Hansen, 1984 |
| Bambusaceae sp. (Bamboo sprouts) | *Pentalagus furnessi* | Poaceae | C3 | Sugimura, 1990 |
| *Barleria* sp. | *Pronolagus crassicaudatus* | Acanthaceae | C3 | Stewart, 1971 |
| *Bassia salsoloides*  (*Kochia pubescens*) | *Bunolagus monticularis* | Chenopodiaceae | C4 | Duthie and Robinson, 1990 |
| *Bastardiastrum gracile* | *Lepus flavigularis* | Malvaceae | C3 | Lorenzo et al., 2011 |
| *Beta vulgaris* | *Lepus europaeus* | Chenopodiaceae | C3 | Reichlin et al., 2006 |
| *Betula glandulosa* | *Lepus timidus* | Betulaceae | C3 | Seccombe-Hett and Turkington, 2008 |
| *Betula populifolia* | *Sylvilagus floridanus* | Betulaceae | C3 | Chapman et al., 1980 |
| *Bignonia capreolata* | *Sylvilagus aquaticus* | Bignoniaceae | C3 | Terrel, 1972 |
| *Bothriochloa saccharoides* | *Sylvilagus auduboni* | Poaceae | C4 | Scribner and Krysl, 1982 |
| *Bouteloua curtipendula* | *Sylvilagus floridanus* | Poaceae | C4 | Hudson et al., 2005 |
| *Bouteloua dactyloides* | *Lepus flavigularis* | Gramineae | C4 | Lorenzo et al., 2011 |
| *Bouteloua gracilis* | *Sylvilagus auduboni* | Poaceae | C4 | Scribner and Krysl, 1982 |
| *Bouteloua repens* | *Lepus flavigularis* | Gramineae | C4 | Lorenzo et al., 2011 |
| *Brassica napus* | *Lepus europaeus* | Brassicaceae | C3 | Katona et al., 2010 |
| *Bromus hordeaceus* | *Lepus europaeus*  *Lepus granatensis* | Poaceae | C3 | Reichlin et al., 2006  Pauperio and Alves., 2008 |
| *Bromus japonicus* | *Lepus europaeus* | Poaceae | C3 | Reichlin et al., 2006 |
| *Bromus* sp. | *Lepus europaeus* | Poaceae | C3 | Katona et al., 2010 |
| *Bromus* sp. | *Lepus europaeus* | Poaceae | C3 | Reichlin et al., 2006 |
| *Bromus sterilis* | *Lepus europaeus* | Poaceae | C3 | Reichlin et al., 2006 |
| *Bromus tectorum* | *Brachylagus idahoensis* | Poaceae | C3 | Green and Flinders, 1980 |
| *Buchloe dactyloides* | *Sylvilagus auduboni* | Poaceae | C4 | Scribner and Krysl, 1982 |
| *Buddleja microphylla* | *Romerolagus diazi* | Loganiaceae | C3 | Cervantes and Martinez, 1992 |
| *Callicarpa japonica* | *Lepus brachyurus* | Verbenaceae | C3 | Shimizu and Shimano, 2010 |
| *Campsis radicans* | *Sylvilagus aquaticus* | Bignoniaceae | C3 | Terrel, 1972 |
| *Capsella bursa pastroris* | *Lepus europaeus* | Brassicaceae | C3 | Katona et al., 2010 |
| *Carex* sp. | *Pentalagus furnessi* | Cyperaceae | C3 | Yamada, 2008 |
| *Carex arenaria* | *Oryctolagus cuniculus* | Cyperaceae | C3 | Alves et al., 2006 |
| *Carex grayii* | *Sylvilagus aquaticus* | Cyperaceae | C3 | Terrel, 1972 |
| *Carex muskingumensis* | *Sylvilagus aquaticus* | Cyperaceae | C3 | Terrel, 1972 |
| *Carex* sp. | *Brachylagus idahoensis* | Cyperaceae | C3 | Green and Flinders, 1980 |
| *Carex* sp. | *Lepus californicus* | Cyperaceae | C3 | Maccracken and Hansen, 1984 |
| *Carex* sp. | *Lepus europaeus* | Cyperaceae | C3 | Katona et al., 2010 |
| *Carex* sp. | *Oryctolagus cuniculus* | Cyperaceae | C3 | Alves et al., 2006 |
| *Carex* sp. | *Sylvilagus nuttalli* | Cyperaceae | C3 | Maccracken and Hansen, 1984 |
| *Carya laciniosa* | *Sylvilagus aquaticus* | Juglandaceae | C3 | Terrel, 1972 |
| **Caryophyllaceae** | *Lepus granatensis* | **Caryophyllaceae** | C3 | Pauperio and Alves, 2008 |
| *Castanea crenata* | *Lepus brachyurus* | Fagaceae | C3 | Shimizu and Shimano, 2010 |
| *Castanopsis sieboldii* | *Pentalagus furnessi* | Fagaceae | C3 | Yamada, 2008 |
| *Castanopsis sieboldii** | *Pentalagus furnessi* | Fagaceae | C3 | Sugimura, 1990 |
| *Castilleja arvensis* | *Sylvilagus floridanus* | Scrophulariaceae | C4 | Hudson et al., 2005 |
| *Cathestechum brevifolium* | *Lepus flavigularis* | Poaceae | C4 | Lorenzo et al., 2011 |
| *Celmisia allanii* | *Lepus europaeus* | Asteraceae | C3 | Flux, 1967 |
| *Celmisia coriacea* | *Lepus europaeus* | Asteraceae | C3 | Flux, 1967 |
| *Celmisia spectabilis* | *Lepus europaeus* | Asteraceae | C3 | Flux, 1967 |
| *Celtis* sp. | *Sylvilagus aquaticus* | Cannabaceae | c3 | Terrel, 1972 |
| *Centrosema pubescens* | *Sylvilagus floridanus* | Fabaceae | C3 | Hudson et al., 2005 |
| *Chaenopodium* sp. | *Lepus europaeus* | Amaranthaceae | C3 | Katona et al., 2010 |
| *Chamaecrista flexuosa* | *Lepus flavigularis* | Fabaceae | C3 | Lorenzo et al., 2011 |
| *Chamaemelium nobile* | *Oryctolagus cuniculus* | Asteraceae | C3 | Alves et al., 2006 |
| *Cheilanthes bonariensis* | *Sylvilagus floridanus* | Adiantaceae | C3 | Hudson et al., 2005 |
| *Chenoleoides tomentosa* | *Oryctolagus cuniculus* | Chenopodiaceae | C4 | Martin et al., 2003 |
| *Chenopodium album* | *Sylvilagus auduboni* | Amaranthaceae | C4 | Scribner and Krysl, 1982 |
| *Chionochloa flavescens* | *Lepus europaeus* | Poaceae | C3 | Flux, 1967 |
| *Chionochloa pallens* | *Lepus europaeus* | Poaceae | C3 | Flux, 1967 |
| *Chionochloa rubra* | *Lepus europaeus* | Poaceae | C3 | Flux, 1967 |
| *Chloris* sp. | *Sylvilagus auduboni* | Poaceae | C4 | Scribner and Krysl, 1982 |
| *Chrysothamnus nauseosus* | *Lepus californicus* | Asteraceae | C3 | Maccracken and Hansen, 1984 |
| *Chrysothamnus viscidiflorus* | *Brachylagus idahoensis* | Asteraceae | C3 | Green and Flinders, 1980 |
| *Cirsium arvense* | *Lepus europaeus* | Asteraceae | C3 | Reichlin et al., 2006 |
| *Cirsium jorullense* | *Romerolagus diazi* | Asteraceae | C3 | Cervantes and Martinez, 1992 |
| *Cirsium* sp. | *Lepus europaeus* | Asteraceae | C3 | Reichlin et al., 2006 |
| *Cistus crispus* | *Oryctolagus cuniculus* | Cistaceae | C3 | Alves et al., 2006 |
| *Cistus salvifolius* | *Oryctolagus cuniculus* | Cistaceae | C3 | Alves et al., 2006 |
| *Clerodendron trichotonum* | *Pentalagus furnessi* | Verbenaceae | C3 | Sugimura, 1990 |
| *Clethra barvinervis* | *Lepus brachyurus* | Clethraceae | C3 | Shimizu and Shimano, 2010 |
| *Cologania* sp. | *Sylvilagus floridanus* | Fabaceae | C3 | Hudson et al., 2005 |
| *Commelina diffusa* | *Sylvilagus floridanus* | Commelinaceae | C3 | Hudson et al., 2005 |
| *Convolvulus arvensis* | *Lepus europaeus* | Convolvulaceae | C3 | Reichlin et al., 2006 |
| *Convolvulus* sp. | *Sylvilagus floridanus* | Convolvulaceae | C3 | Hudson et al., 2005 |
| *Conyza canadensis* | *Sylvilagus auduboni* | Asteraceae | C3 | Scribner and Krysl, 1982 |
| *Conyza rouyana* | *Oryctolagus cuniculus* | Asteraceae | C3 | Alves et al., 2006 |
| *Coprosma brunnea* | *Lepus europaeus* | Rubiaceae | C3 | Flux, 1967 |
| *Coprosoma pseudocuneata* | *Lepus europaeus* | Rubiaceae | C3 | Flux, 1967 |
| *Corema album* | *Oryctolagus cuniculus* | Ericaceae | C3 | Alves et al., 2006  Larrinaga, 2010 |
| *Cornus sanguinea* | *Lepus europaeus* | Cornaceae | C3 | Reichlin et al., 2006 |
| *Corylus sieboldiana* | *Lepus brachyurus* | Betulaceae | C3 | Shimizu and Shimano, 2010 |
| *Corynephorus canescens* | *Lepus granatensis*  *Oryctolagus cuniculus* | Poaceae | C3 | Alves et al., 2006  Pauperio and Alves, 2008 |
| *Crepis* sp. | *Lepus europaeus* | Rosaceae | C3 | Katona et al., 2010 |
| *Crotalaria* sp. | *Pronolagus crassicaudatus* | Asteraceae | C3 | Stewart, 1971 |
| *Croton texansis* | *Sylvilagus auduboni* | Euphorbiaceae | C3 | Scribner and Krysl, 1982 |
| *Cryptomeria japonica* | *Lepus brachyurus* | Cupressaceae | C3 | Shimizu and Shimano, 2010 |
| *Cymbopogon* sp. | *Caprolagus hispidus* | Poaceae | C4 | Bell et al., 1990 |
| *Cymbopogon* sp. | *Caprolagus hispidus* | Poaceae | C4 | Yadav et al., 2008 |
| *Cynodon dactylon* | *Lepus europaeus* | Poaceae | C4 | Katona et al., 2010 |
| *Cynosurus echinatus* | *Lepus granatensis* | Poaceae | C3 | Pauperio and Alves, 2008 |
| *Cyperus arsenii* | *Sylvilagus floridanus* | Cyperaceae | C3 | Hudson et al., 2005 |
| *Cyperus reflexa* | *Sylvilagus floridanus* | Cyperaceae | C3 | Hudson et al., 2005 |
| *Cyperus semiochraceus* | *Lepus flavigularis* | Cyperaceae | C4 | Lorenzo et al., 2011 |
| *Cyrsium* sp. | *Romerolagus diazi* | Asteraceae | C3 | Fa and Bell, 1990 |
| *Cyrtandra* sp. | *Nesolagus netscheri* | Gesneriaceae | C3 | Flux, 1990 |
| *Cytisus grandiﬂorus* | *Oryctolagus cuniculus* | Fabaceae | C3 | Alves et al., 2006 |
| *Dactylis glomerata* | *Lepus europaeus*  *Lepus granatensis*  *Sylvilagus floridanus*  *Oryctolagus cuniculus* | Poaceae | C3 | Chapman et al., 1980 ;  Alves et al., 2006 ;  Reichlin et al., 2006 ;  Pauperio and Alves., 2008 |
| *Dactyloctenium aegyptium* | *Lepus flavigularis* | Poaceae | C4 | Lorenzo et al., 2011 |
| *Daucus carota* | *Lepus europaeus*  *Sylvilagus floridanus* | Apiaceae | C3 | Reichlin et al., 2006 |
| *Descurainia pinnata* | *Sylvilagus nuttalli* | Brassicaceae | C3 | Maccracken and Hansen, 1984 |
| *Desmodium densiflorum* | *Sylvilagus floridanus* | Fabaceae | C3 | Hudson et al., 2005 |
| *Digitaria ciliaris* | *Lepus flavigularis* | Poaceae | C4 | Lorenzo et al., 2011 |
| *Dracophyllum uniflorum* | *Lepus europaeus* | Ericaceae | C3 | Flux, 1967 |
| *Dyschoriste microphylla* | *Sylvilagus floridanus* | Acanthaceae | C3 | Hudson et al., 2005 |
| *Echinochloa crusgalli* | *Sylvilagus auduboni* | Poaceae | C4 | Scribner and Krysl, 1982 |
| *Elaeagnus angustifolia* | *Lepus europaeus* | Elaeagnaceae | C3 | Katona et al., 2010 |
| *Elatostemma* sp. | *Nesolagus netscheri* | Rubiaceae | C3 | Flux, 1990 |
| *Eleocharis* sp. | *Sylvilagus auduboni* | Cyperaceae | Unknown* | Scribner and Krysl, 1982 |
| *Elymus canadensis* | *Sylvilagus floridanus* | Poaceae | C3 | Chapman et al., 1980 |
| *Elymus farctus* | *Oryctolagus cuniculus* | Poaceae | C3 | Alves et al., 2006 |
| *Elymus virginicus* | *Sylvilagus aquaticus* | Poaceae | C3 | Terrel, 1972 |
| *Epicampes* sp. | *Romerolagus diazi* | Poaceae | C3 | Fa and Bell, 1990 |
| *Epilobium angustifolium* | *Lepus timidus* | Onagraceae | C3 | Seccombe-Hett and Turkington, 2008 |
| *Eragrostis intermedia* | *Lepus flavigularis* | Poaceae | C4 | Lorenzo et al., 2011 |
| *Eragrostis mexicana* | *Sylvilagus floridanus* | Poaceae | C4 | Hudson et al., 2005 |
| *Eragrostis pilosa* | *Lepus flavigularis* | Poaceae | C4 | Lorenzo et al., 2011 |
| *Erianthum ravennae* | *Caprolagus hispidus* | Poaceae | C3 | Bell et al., 1990 |
| *Erica* sp. | *Lepus granatensis* | Ericaceae | C3 | Pauperio and Alves, 2008 |
| *Erigeron* sp. | *Lepus californicus* | Asteraceae | C3 | Maccracken and Hansen, 1984 |
| *Erigeron* sp. | *Sylvilagus nuttalli* | Asteraceae | C3 | Maccracken and Hansen, 1984 |
| *Eriogonum heracleoides* | *Brachylagus idahoensis* | Polygonaceae | C3 | Green and Flinders, 1980 |
| *Erioneuron pilosum* | *Sylvilagus auduboni* | Poaceae | C4 | Scribner and Krysl, 1982 |
| *Eryngium columnare* | *Romerolagus diazi* | Apiaceae | C3 | Cervantes and Martinez, 1992 |
| *Erynigium* sp. | *Romerolagus diazi* | Apiaceae | C3 | Fa and Bell, 1990 |
| *Euphorbia* sp. | *Sylvilagus floridanus* | Euphorbiaceae | Unknow* | Hudson et al., 2005 |
| *Eurotia lanata* | *Lepus californicus*  *Sylvilagus nuttalli* | **Chenopodiaceae** | C3 | Maccracken and Hansen, 1984 |
| *Evonymus atropurpureus* | *Sylvilagus aquaticus* | Celastraceae | C3 | Terrel, 1972 |
| *Fagopyrum esculentum* | *Lepus europaeus* | Polygonaceae | C3 | Reichlin et al., 2006 |
| *Festuca altaica* | *Lepus timidus* | Poaceae | C3 | Seccombe-Hett and Turkington, 2008 |
| *Festuca amplissima* | *Romerolagus diazi* | Poaceae | C3 | Cervantes and Martinez, 1992 |
| *Festuca* sp. | *Lepus europaeus* | Poaceae | C3 | Katona et al., 2010 |
| *Festuca* sp. | *Lepus granatensis* | Poaceae | C3 | Pauperio and Alves, 2008 |
| *Festuca* sp. | *Oryctolagus cuniculus* | Poaceae | C3 | Alves et al., 2006 |
| *Ficus erecta* | *Pentalagus furnessi* | Moraceae | C3 | Sugimura, 1990 |
| *Fraxinus sieboldiana* | *Lepus brachyurus* | Oleaceae | C3 | Shimizu and Shimano, 2010 |
| *Fraxinus* sp. | *Sylvilagus aquaticus* | Oleaceae | C3 | Terrel, 1972 |
| *Fuchsia thymifolia* | *Romerolagus diazi* | Onagraceae | C3 | Cervantes and Martinez, 1992 |
| *Furcraea bedinghausii* | *Romerolagus diazi* | Amaryllidaceae | C3 | Cervantes and Martinez, 1992 |
| *Galium obtusum* | *Sylvilagus aquaticus* | Rubiaceae | C3 | Terrel, 1972 |
| *Galium verum* | *Lepus europaeus* | Rubiaceae | C3 | Reichlin et al., 2006 |
| *Gardenia jasminoides* | *Pentalagus furnessi* | Rubiaceae | C3 | Sugimura, 1990 |
| *Gaultheria depressa* | *Lepus europaeus* | Ericaceae | C3 | Flux, 1967 |
| *Gaultheria procumbens* | *Sylvilagus obscurus* | Ericaceae | C3 | Hartman and Barry, 2010 |
| *Gaylussacia baccata* | *Sylvilagus obscurus* | Ericaceae | C3 | Hartman and Barry, 2010 |
| *Geranium* sp. | *Romerolagus diazi* | Geraniaceae | C3 | Cervantes and Martinez, 1992 |
| *Gleditsia* sp. | *Sylvilagus aquaticus* | Fabaceae | C3 | Terrel, 1972 |
| *Gleditsia* sp. (seed) | *Lepus europaeus* | Fabaceae | C3 | Katona et al., 2010 |
| *Glycine max* | *Lepus europaeus* | Fabaceae | C3 | Reichlin et al., 2006 |
| *Gnaphalium liebmannii* | *Sylvilagus floridanus* | Asteraceae | C3 | Hudson et al., 2005 |
| *Halimium* sp. | *Oryctolagus cuniculus* | Cistaceae | C3 | Alves et al., 2006 |
| *Halogeton glomeratus* | *Lepus californicus*  *Sylvilagus nuttalli* | Chenopodiaceae | C4 | Maccracken and Hansen, 1984 |
| *Hamamelis virginiana* | *Sylvilagus obscurus* | Hamamelidaceae | C3 | Hartman and Barry, 2010 |
| *Hebe pauciramosa* | *Lepus europaeus* | Plantaginaceae | C3 | Flux, 1967 |
| *Helianthemum aegyptiacum* | *Lepus granatensis* | Cistaceae | C3 | Pauperio and Alves, 2008 |
| *Helianthus annuus* | *Sylvilagus auduboni* | Asteraceae | C3 | Scribner and Krysl, 1982 |
| *Helianthus ciliaris* | *Sylvilagus auduboni* | Asteraceae | C3 | Scribner and Krysl, 1982 |
| *Helianthus onnuus* | *Sylvilagus auduboni* | Asteraceae | C3 | Scribner and Krysl, 1982 |
| *Helichrysum selago* | *Lepus europaeus* | Asteraceae | C3 | Flux, 1967 |
| *Helictotrichon pubescens* | *Lepus europaeus* | Poaceae | C3 | Reichlin et al., 2006 |
| *Heliotropium ramosissimum* | *Oryctolagus cuniculus* | Boraginaceae | C4 | Martin et al., 2003 |
| *Helwingia japonica* | *Lepus brachyurus* | Helwingiaceae | C3 | Shimizu and Shimano, 2010 |
| *Hemigraphis colorata* | *Nesolagus netscheri* | Acanthaceae | C3 | Flux, 1990 |
| *Heteropogon contorius* | *Pronolagus crassicaudatus* | Poaceae | C4 | Stewart, 1971 |
| *Hibiscus* sp. | *Pronolagus crassicaudatus* | Malvaceae | C3 | Stewart, 1971 |
| *Hilaria cenchroides* | *Sylvilagus floridanus* | Poaceae | C4 | Hudson et al., 2005 |
| *Holcus annus* | *Lepus granatensis* | Poaceae | C3 | Pauperio and Alves, 2008 |
| *Holcus lanatus* | *Oryctolagus cuniculus*  *Lepus europaeus*  *Lepus granatensis* | Poaceae | C3 | Flux, 1967;  Alves et al., 2006 |
| *Holcus* sp. | *Lepus granatensis* | Poaceae | C3 | Pauperio and Alves, 2008 |
| *Hordeum murinum* | *Lepus europaeus*  *Lepus granatensis* | Poaceae | C3 | Reichlin et al., 2006  Pauperio and Alves., 2008 |
| *Hordeum vulgare* | *Lepus europaeus* | Poaceae | C3 | Reichlin et al., 2006 |
| *Hydrangea serrata* | *Lepus brachyurus* | Hydrangeaceae | C3 | Shimizu and Shimano, 2010 |
| *Hymenanthera alpina* | *Lepus europaeus* | Violaceae | C3 | Flux, 1967 |
| *Hyparrhenia* sp. | *Pronolagus crassicaudatus* | Poaceae | C4 | Stewart, 1971 |
| *Hypochaeris radicata* | *Lepus granatensis* | Asteraceae | C3 | Pauperio and Alves, 2008 |
| *Ilex verticillata* | *Sylvilagus floridanus* | Aquifoliaceae | C3 | Chapman et al., 1980 |
| *Imperata cylindrica* | *Caprolagus hispidus* | Gramineae | C4 | Bell et al., 1990  Yadav et al., 2008 |
| *Ipomea wrightii* | *Lepus flavigularis* | Convulvolaceae | C3 | Lorenzo et al., 2011 |
| *Ipomoea batatas* | *Nesolagus netscheri*  *Pentalagus furnessi* | Convulvolaceae | C3 | Flux, 1990  Sugimura, 1990 |
| *Ischaemum afrum* | *Pronolagus crassicaudatus* | Poaceae | C4 | Stewart, 1971 |
| *Juncus capitatus* | *Lepus europaeus* | Juncaceae | C3 | Reichlin et al., 2006 |
| *Juncus* sp. | *Lepus europaeus* | Juncaceae | C3 | Reichlin et al., 2006 |
| *Juncus* sp. | *Oryctolagus cuniculus* | Juncaceae | C3 | Alves et al., 2006 |
| *Juniperus deppeana* | *Sylvilagus floridanus* | Cupressaceae | C3 | Hudson et al., 2005 |
| *Jussieua suffruticosa* | *Nesolagus netscheri* | Onagraceae | C3 | Flux, 1990 |
| *Kochia scoparia* | *Sylvilagus auduboni* | Chenopodiaceae | C4 | Scribner and Krysl, 1982 |
| *Koeleria cristata* | *Brachylagus idahoensis* | Poaceae | C3 | Green and Flinders, 1980 |
| *Lactuca scariola* | *Sylvilagus floridanus* | Asteraceae | C3 | Chapman et al., 1980 |
| *Lamiaceae* sp. | *Romerolagus diazi* | Lamiaceae | C3 | Cervantes and Martinez, 1992 |
| *Lamium purpureum* | *Lepus europaeus* | Lamiaceae | C3 | Reichlin et al., 2006 |
| *Laportea canadensis* | *Sylvilagus aquaticus* | Urticaceae | C3 | Terrel, 1972 |
| *Lathyrus sativus* | *Lepus europaeus* | Faboideae | C3 | Reichlin et al., 2006 |
| *Launaea arborescens* | *Oryctolagus cuniculus* | Asteraceae | C3 | Martin et al., 2003 |
| *Lavandula sampaiana* | *Oryctolagus cuniculus* | Lamiaceae | C3 | Alves et al., 2006 |
| *Lea crispa* | *Caprolagus hispidus* | Leeaceae | C3 | Bell et al., 1990 |
| *Lespedeza cyrtobotrya* | *Lepus brachyurus* | Fabaceae | C3 | Shimizu and Shimano, 2010 |
| *Lespedeza stipulacea* | *Sylvilagus floridanus* | Fabaceae | C3 | Chapman et al., 1980 |
| *Liliaceae* sp. | *Lepus granatensis* | Liliaceae | C3 | Pauperio and Alves, 2008 |
| *Lindera umbellata var.membranacea* | *Lepus brachyurus* | Lauraceae | C3 | Shimizu and Shimano, 2010 |
| *Lonicera* sp. | *Sylvilagus obscurus* | Caprifoliaceae | C3 | Hartman and Barry, 2010 |
| *Lotus corniculatus* | *Lepus europaeus* | Faboideae | C3 | Reichlin et al., 2006 |
| *Lupinus arcticus* | *Lepus timidus* | Fabaceae | C3 | Seccombe-Hett and Turkington, 2008 |
| *Lupinus sp.* | *Brachylagus idahoensis* | Fabaceae | C3 | Green and Flinders, 1980 |
| *Luzula lactea* | *Lepus granatensis* | Juncaceae | C3 | Pauperio and Alves, 2008 |
| *Lycium intricatum* | *Oryctolagus cuniculus* | Solanaceae | C4 | Martin et al., 2003 |
| *Lycurus phalaroides* | *Sylvilagus floridanus* | Poaceae | C4 | Hudson et al., 2005 |
| *Lyonia ligustrina* | *Sylvilagus floridanus* | Ericaceae | C3 | Chapman et al., 1980 |
| *Mallotus japonicus* | *Lepus brachyurus*  *Pentalagus furnessi* | Euphorbiaceae | C3 | Shimizu and Shimano, 2010  Sugimura, 1990 |
| *Malus domestica* | *Lepus europaeus* | Rosaceae | C3 | Reichlin et al., 2006 |
| *Malus pumila* | *Sylvilagus floridanus* | Rosaceae | C3 | Chapman et al., 1980 |
| *Medicago sativa* | *Lepus europaeus* | Fabaceae | C3 | Reichlin et al., 2006  Katona et al., 2010 |
| *Melastoma candidum* | *Pentalagus furnessi* | Melastomataceae | C3 | Yamada, 2008 |
| *Menispermum canadense* | *Sylvilagus aquaticus* | Menispermaceae | C3 | Terrel, 1972 |
| *Mercurialis annua* | *Oryctolagus cuniculus* | Euphorbiaceae | C3 | Martin et al., 2003 |
| *Mertensia* sp. | *Sylvilagus nuttalli* | Boraginaceae | C3 | Maccracken and Hansen, 1984 |
| *Mesembryanthemaceae* sp. | *Bunolagus monticularis* | Aizoaceae | C3 | Duthie and Robinson, 1990 |
| *Mesembryanthemum crystallinum* | *Oryctolagus cuniculus* | Aizoaceae | C3 | Martin et al., 2003 |
| *Mesembryanthemum nodiflorum* | *Oryctolagus cuniculus* | Aizoaceae | C3 | Martin et al., 2003 |
| *Mesembryanthemum* sp. | *Oryctolagus cuniculus* | Aizoaceae | C3 | Martin et al., 2003 |
| *Microchloa kunthii* | *Sylvilagus floridanus* | Poaceae | C4 | Hudson et al., 2005 |
| *Micropyrum* sp. | *Lepus granatensis* | Poaceae | C3 | Pauperio and Alves, 2008 |
| *Mimosa tenuiflora* | *Lepus flavigularis* | Fabaceae | C3 | Lorenzo et al., 2011 |
| *Miscanthus sinensis* | *Pentalagus furnessi* | Gramineae | C4 | Sugimura, 1990  Yamada, 2008 |
| *Mosla dianthera* | *Pentalagus furnessi* | Lamiaceae | C3 | Yamada, 2008 |
| *Muehlenbeckia axillaris* | *Lepus europaeus* | Polygonaceae | C3 | Flux, 1967 |
| *Muhlenbergia implicata* | *Sylvilagus floridanus* | Poaceae | C4 | Hudson et al., 2005 |
| *Muhlenbergia macroura* | *Romerolagus diazi* | Poaceae | C4 | Cervantes and Martinez, 1992 |
| *Muhlenbergia microsperma* | *Lepus flavigularis* | Poaceae | C4 | Lorenzo et al., 2011 |
| *Murinum* sp. | *Lepus granatensis* | Poaceae | C3 | Pauperio and Alves, 2008 |
| *Museniopsis arguta* | *Romerolagus diazi* | Umbelliferae | C3 | Fa and Bell, 1990 |
| *Mussaeenda parviflora* | *Pentalagus furnessi* | Rubiaceae | C3 | Sugimura, 1990 |
| *Narenga porphyrocoma* | *Caprolagus hispidus* | Poaceae | C4 | Yadav et al., 2008 |
| *Narenga* sp. | *Caprolagus hispidus* | Poaceae | C4 | Bell et al., 1990 |
| *Nothofagus solandri var. cliffortioides* | *Lepus europaeus* | Nothofagaceae | C3 | Flux, 1967 |
| *Opuntia polyacantha* | *Lepus californicus,*  *Sylvilagus nuttalli* | Cactaceae | C3 | Maccracken and Hansen, 1984 |
| *Oreomyrrhis colensoi* | *Lepus europaeus* | Apiaceae | C3 | Flux, 1967 |
| *Oryzopsis hymenoides* | *Brachylagus idahoensis*  *Lepus californicus*  *Sylvilagus nuttalli* | Poaceae | C3 | Green and Flinders, 1980;  Maccracken and Hansen, 1984 |
| *Osteospermum spinescens* | *Bunolagus monticularis* | Asteraceae | C3 | Duthie et al., 1989 |
| *Oxalis alpina* | *Sylvilagus floridanus* | Oxalidaceae | C3 | Hudson et al., 2005 |
| *Oxalis corniculata* | *Sylvilagus floridanus* | Oxalidaceae | C3 | Hudson et al., 2005 |
| *Panicum miliaceum* | *Lepus europaeus* | Poaceae | C4 | Reichlin et al., 2006 |
| *Panicum obtusutm* | *Sylvilagus auduboni* | Poaceae | C4 | Scribner and Krysl, 1982 |
| *Papaver rhoeas* | *Lepus europaeus* | Papaveraceae | C3 | Reichlin et al., 2006 |
| *Paspalum notatum* | *Lepus flavigularis* | Poaceae | C4 | Lorenzo et al., 2011 |
| *Paspalum postratum* | *Sylvilagus floridanus* | Poaceae | C4 | Hudson et al., 2005 |
| *Patellifolia patellaris* | *Oryctolagus cuniculus* | Chenopodiaceae | C3 | Martin et al., 2003 |
| *Penstemon* sp. | *Brachylagus idahoensis* | Plantaginaceae | C3 | Green and Flinders, 1980 |
| *Peucedanum japonicum* | *Pentalagus furnessi* | Apiaceae | C3 | Yamada, 2008 |
| *Phacelia tanacetifolia* | *Lepus europaeus* | Boraginaceae | C3 | Reichlin et al., 2006 |
| *Phaseolus heterophyllus* | *Sylvilagus floridanus* | Fabaceae | C3 | Hudson et al., 2005 |
| *Phleum pratense* | *Sylvilagus floridanus* | Poaceae | C3 | Chapman et al., 1980 |
| *Photinia* sp. | *Sylvilagus obscurus* | Rosaceae | C3 | Hartman and Barry, 2010 |
| *Phragmites australis* | *Oryctolagus cuniculus* | Poaceae | C3 | Alves et al., 2006 |
| *Phragmites* sp. | *Caprolagus hispidus* | Poaceae | C3 | Bell et al., 1990 |
| *Physalis mollis* | *Romerolagus diazi* | Solanaceae | C3 | Cervantes and Martinez, 1992 |
| *Phytolacca* sp. | *Sylvilagus floridanus* | Phytolaccaceae | C3 | Hudson et al., 2005 |
| *Pinus densiflora* | *Lepus brachyurus* | Pinaceae | C3 | Shimizu and Shimano, 2010 |
| *Pinus pinaster* | *Oryctolagus cuniculus* | Pinaceae | C3 | Alves et al., 2006 |
| *Pinus* sp. | *Lepus europaeus* | Pinaceae | C3 | Katona et al., 2010 |
| *Piptochaetium fimbriatum* | *Sylvilagus floridanus* | Poaceae | C3 | Hudson et al., 2005 |
| *Piptochaetium seleri* | *Sylvilagus floridanus* | Poaceae | C3 | Hudson et al., 2005 |
| *Piptochaetium virescens* | *Sylvilagus floridanus* | Poaceae | C3 | Hudson et al., 2005 |
| *Pisum sativum* | *Lepus europaeus* | Fabaceae | C3 | Reichlin et al., 2006 |
| *Pittosporum divaricatum* | *Lepus europaeus* | Pittosporaceae | C3 | Flux, 1967 |
| *Plantago lanceolata* | *Lepus europaeus* | Plantaginaceae | C3 | Reichlin et al., 2006 |
| *Plantago linearis* | *Sylvilagus floridanus* | Plantaginaceae | C3 | Hudson et al., 2005 |
| *Plantago rugelii* | *Sylvilagus floridanus* | Plantaginaceae | C3 | Chapman et al., 1980 |
| *Poa annua* | *Lepus europaeus* | Poaceae | C3 | Reichlin et al., 2006 |
| *Poa bulbosa* | *Lepus granatensis* | Poaceae | C3 | Pauperio and Alves, 2008 |
| *Poa colensoi* | *Lepus europaeus* | Poaceae | C3 | Flux, 1967 |
| *Poa nevadensis* | *Brachylagus idahoensis* | Poaceae | C3 | Green and Flinders, 1980 |
| *Poa* sp. | *Lepus californicus* | Poaceae | C3 | Maccracken and Hansen, 1984 |
| *Poa* sp. | *Oryctolagus cuniculus* | Poaceae | C3 | Alves et al., 2006 |
| *Poa* sp. | *Sylvilagus nuttalli* | Poaceae | C3 | Maccracken and Hansen, 1984 |
| *Podocarpus macrophyllus* | *Pentalagus furnessi* | Podocarpaceae | C3 | Sugimura, 1990 |
| *Poe compressa* | *Sylvilagus floridanus* | Poaceae | C3 | Chapman et al., 1980 |
| *Poe pratense* | *Sylvilagus floridanus* | Poaceae | C3 | Chapman et al., 1980 |
| *Polygala serpyllifolia* | *Lepus granatensis* | Polygalaceae | C4 | Pauperio and Alves, 2008 |
| *Polygala* sp. | *Lepus granatensis* | Polygalaceae | C4 | Pauperio and Alves, 2008 |
| *Polygonum aviculare* | *Lepus europaeus* | Polygonaceae | C3 | Reichlin et al., 2006 |
| *Polygonum* sp. | *Nesolagus netscheri* | Polygonaceae | C3 | Flux, 1990 |
| *Populus balsamifera* | *Lepus americanus* | Salicaceae | C3 | Schmitz et al., 1992 |
| *Populus tremuloides* | *Sylvilagus floridanus* | Salicaceae | C3 | Chapman et al., 1980 |
| *Prunnus spinosa* | *Lepus europaeus* | Rosaceae | C3 | Katona et al., 2010 |
| *Prunus serotina* | *Sylvilagus floridanus* | Rosaceae | C3 | Chapman et al., 1980 |
| *Prunus verecunda* | *Lepus brachyurus* | Rosaceae | C3 | Shimizu and Shimano, 2010 |
| *Prunus virginiana* | *Sylvilagus floridanus* | Rosaceae | C3 | Chapman et al., 1980 |
| *Psilocaulon coriarium* | *Bunolagus monticularis* | Aizoaceae | C3 | Duthie et al., 1989 |
| *Psychotria serpens* | *Pentalagus furnessi* | Rubiaceae | C3 | Sugimura, 1990 |
| *Pteronia erythrocaetha* | *Bunolagus monticularis* | Begoniaceae | C3 | Duthie and Robinson, 1990 |
| *Purshia tridentate* | *Brachylagus idahoensis* | Rosaceae | C3 | Green and Flinders, 1980 |
| *Quercus serrata* | *Lepus brachyurus* | Fagaceae | C3 | Shimizu and Shimano, 2010 |
| *Quercus* sp. | *Sylvilagus floridanus* | Fagaceae | C3 | Hudson et al., 2005 |
| *Ranunculus* sp. | *Sylvilagus aquaticus* | Ranunculaceae | C3 | Terrel, 1972 |
| *Retama monosperma* | *Lepus granatensis* | Fabaceae | C3 | Pauperio and Alves, 2008 |
| *Rhus radicans* | *Sylvilagus aquaticus* | Anacardiaceae | C3 | Terrel, 1972 |
| *Rhus trichocarpa* | *Lepus brachyurus* | Anacardiaceae | C3 | Shimizu and Shimano, 2010 |
| *Rhus typhina* | *Sylvilagus floridanus* | Anacardiaceae | C3 | Chapman et al., 1980 |
| *Ribes affine* | *Romerolagus diazi* | Saxifragaceae | C3 | Cervantes and Martinez, 1992 |
| *Ribes* sp. | *Brachylagus idahoensis* | Saxifragaceae | C3 | Green and Flinders, 1980 |
| *Rinanthus minor* | *Lepus granatensis* | Orobanchaceae | C3 | Pauperio and Alves, 2008 |
| *Rosenia humilis* | *Bunolagus monticularis* | Asteraceae | C3 | Duthie and Robinson, 1990 |
| *Rubus allegheniensis* | *Sylvilagus floridanus* | Rosaceae | C3 | Chapman et al., 1980 |
| *Rubus argutus* | *Sylvilagus aquaticus* | Rosaceae | C3 | Terrel, 1972 |
| *Rubus caesius* | *Lepus europaeus* | Rosaceae | C3 | Reichlin et al., 2006 |
| *Rubus crataegifolius* | *Lepus brachyurus* | Rosaceae | C3 | Shimizu and Shimano, 2010 |
| *Rubus palmatus var.coptophyllus* | *Lepus brachyurus* | Rosaceae | C3 | Shimizu and Shimano, 2010 |
| *Rubus sieboldii* | *Pentalagus furnessi* | Rosaceae | C3 | Yamada, 2008 |
| *Rubus* sp. | *Lepus europaeus* | Rosaceae | C3 | Katona et al., 2010 |
| *Rubus strigosus* | *Sylvilagus floridanus* | Rosaceae | C3 | Chapman et al., 1980 |
| *Rubus villosus* | *Sylvilagus floridanus* | Rosaceae | C3 | Chapman et al., 1980 |
| *Rumex acetosella subsp.* | *Lepus granatensis* | Polygonaceae | C3 | Pauperio and Alves, 2008 |
| *Rumex crispus* | *Sylvilagus floridanus*  *Sylvilagus auduboni* | Polygonaceae | C3 | Chapman et al., 1980 |
| *Saccharum munja* | *Caprolagus hispidus* | Poaceae | C4 | Bell et al., 1990 |
| *Saccharum* sp. | *Caprolagus hispidus* | Poaceae | C4 | Bell et al., 1990 |
| *Saccharum spontaneum* | *Caprolagus hispidus* | Poaceae | C4 | Bell et al., 1990 |
| *Salix bakko* | *Lepus brachyurus* | Salicaceae | C3 | Shimizu and Shimano, 2010 |
| *Salix glauca* | *Lepus americanus* | Salicaceae | C3 | Schmitz et al., 1992 |
| *Salix* sp. | *Lepus timidus* | Salicaceae | C3 | Seccombe-Hett and Turkington, 2008 |
| *Salix* sp. | *Sylvilagus floridanus* | Salicaceae | C3 | Chapman et al., 1980 |
| *Salsola divaricata* | *Oryctolagus cuniculus* | Chenopodiaceae | C4 | Martin et al., 2003 |
| *Salsola glabrescens* | *Bunolagus monticularis* | Chenopodiaceae | C4 | Duthie and Robinson, 1990 |
| *Salsola tetranda* | *Oryctolagus cuniculus* | Chenopodiaceae | C4 | Martin et al., 2003 |
| *Salsola vermiculata* | *Oryctolagus cuniculus* | Chenopodiaceae | C4 | Martin et al., 2003 |
| *Salvia elegans* | *Romerolagus diazi* | Lamiaceae | C3 | Cervantes and Martinez, 1992 |
| *Salvia* sp. | *Romerolagus diazi* | Lamiaceae | C3 | Cervantes and Martinez, 1992 |
| *Sambucus* sp. | *Lepus europaeus* | Adoxaceae | C3 | Katona et al., 2010 |
| *Schoenus pauciflorus* | *Lepus europaeus* | Cyperaceae | C3 | Flux, 1967 |
| *Scirpus* sp. | *Sylvilagus auduboni* | Cyperaceae |  | Scribner and Krysl, 1982 |
| *Scutellaria coerulea* | *Romerolagus diazi* | Lamiaceae | C3 | Cervantes and Martinez, 1992 |
| *Secale cereale* | *Lepus europaeus*  *Lepus granatensis* | Poaceae | C3 | Reichlin et al., 2006 |
| *Secale sylvestre* | *Lepus europaeus* | Poaceae | C3 | Katona et al., 2010 |
| *Selaginella pallescens* | *Sylvilagus floridanus* | Selaginellaceae | C3 | Hudson et al., 2005 |
| *Senecio stoechadiformis* | *Romerolagus diazi* | Asteraceae | C3 | Cervantes and Martinez, 1992 |
| *Seseli tortosum* | *Oryctolagus cuniculus* | **Apiaceae** | C3 | Alves et al., 2006 |
| *Setaria* sp. | *Sylvilagus floridanus* | Poaceae | C4 | Hudson et al., 2005 |
| *Shepherdia canadensis* | *Lepus timidus* | Elaeagnaceae | C3 | Seccombe-Hett and Turkington, 2008 |
| *Sicyos coccinea* | *Romerolagus diazi* | Cucurbitaceae | C3 | Cervantes and Martinez, 1992 |
| *Sinapis arvensis** | *Lepus europaeus* | Brassicaceae | C3 | Reichlin et al., 2006 |
| *Smilax* sp. | *Sylvilagus aquaticus* | Smilacaceae | C3 | Terrel, 1972 |
| *Solanum elaeognifolium* | *Sylvilagus auduboni* | Solanaceae | C3 | Scribner and Krysl, 1982 |
| *Solanum* sp. | *Pronolagus crassicaudatus* | Solanaceae | C3 | Stewart, 1971 |
| *Solidago multiradiata* | *Lepus timidus* | Asteraceae | C3 | Seccombe-Hett and Turkington, 2008 |
| *Sorghum halepense* | *Sylvilagus auduboni* | Poaceae | C4 | Scribner and Krysl, 1982 |
| *Spergularia fallax* | *Oryctolagus cuniculus* | Caryophyllaceae | C3 | Martin et al., 2003 |
| *Sphaeralcea coccinea* | *Sylvilagus auduboni* | Malvaceae | C3 | Scribner and Krysl, 1982 |
| *Sporobolus cryptandrus* | *Sylvilagus auduboni* | Poaceae | C4 | Scribner and Krysl, 1982 |
| *Sporobolus indicus* | *Sylvilagus floridanus* | Poaceae | C4 | Hudson et al., 2005 |
| *Stachyurus praecox* | *Lepus brachyurus* | Stachyuraceae | C3 | Shimizu and Shimano, 2010 |
| *Staphylea bumalda* | *Lepus brachyurus* | Staphyleaceae | C3 | Shimizu and Shimano, 2010 |
| *Stellaria media* | *Lepus europaeus* | Caryophyllaceae | C3 | Reichlin et al., 2006 |
| *Stevia serrata* | *Sylvilagus floridanus* | Asteraceae | C3 | Hudson et al., 2005 |
| *Stipa comata* | *Brachylagus idahoensis*  *Lepus californicus*  *Sylvilagus nuttalli* | Poaceae | C3 | Green and Flinders, 1980  Maccracken and Hansen, 1984 |
| *Stipa ichu* | *Sylvilagus floridanus*  *Romerolagus diaz* | Poaceae | C3 | Hudson et al., 2005  Cervantes and Martinez., 1992 |
| *Styrax japonica* | *Pentalagus furnessi* | Styracaceae | C3 | Yamada, 2008 |
| *Suaeda vera* | *Oryctolagus cuniculus* | Chenopodiaceae | C3 | Martin et al., 2003 |
| *Swida controversa* | *Lepus brachyurus* | Cornaceae | C3 | Shimizu and Shimano, 2010 |
| *Synedrella nodiflora* | *Nesolagus netscheri* | Asteraceae | C3 | Flux, 1990 |
| *Taraxacum officinale* | *Lepus europaeus*  *Sylvilagus floridanus* | Asteraceae | C3 | Chapman et al., 1980  Reichlin et al., 2006 |
| *Themeda triandra* | *Pronolagus crassicaudatus* | Poaceae | C4 | Stewart, 1971 |
| *Themeda villosa* | *Caprolagus hispidus* | Poaceae | C4 | Bell et al., 1990 |
| *Thymus serpyllum* | *Lepus europaeus* | Lamiaceae | C3 | Reichlin et al., 2006 |
| *Tradescantia* sp. | *Nesolagus netscheri* | Commelinaceae | C3 | Flux, 1990 |
| *Trichodiadema barbatum* | *Bunolagus monticularis* | Aizoaceae | C3 | Duthie et al., 1989 |
| *Trifolium incarnatus* | *Lepus europaeus* | Fabaceae | C3 | Reichlin et al., 2006 |
| *Trifolium pratense* | *Lepus europaeus*  *Sylvilagus floridanus* | Fabaceae | C3 | Chapman et al., 1980  Reichlin et al., 2006 |
| *Trifolium repens* | *Lepus europaeus* | Fabaceae | C3 | Flux, 1967  Reichlin et al., 2006 |
| *Trifolium* sp. | *Nesolagus netscheri* | Fabaceae | C3 | Flux, 1990 |
| *Trifolium* sp. | *Oryctolagus cuniculus* | Fabaceae | C3 | Alves et al., 2006 |
| *Trifolium suaveolens* | *Lepus europaeus* | Fabaceae | C3 | Reichlin et al., 2006 |
| *Triticum aestivum* | *Lepus europaeus* | Poaceae | C3 | Reichlin et al., 2006 |
| *Typha elephantum* | *Caprolagus hispidus* | Typhaceae | C3 | Bell et al., 1990 |
| *Ulex europaeus* | *Oryctolagus cuniculus* | Fabaceae | C3 | Alves et al., 2006 |
| *Ulex minor* | *Lepus granatensis* | Fabaceae | C3 | Pauperio and Alves, 2008 |
| *Urochloa meziana* | *Lepus flavigularis* | Poaceae | C4 | Lorenzo et al., 2011 |
| *Vaccinium corymbosum* | *Sylvilagus floridanus* | Ericaceae | C3 | Chapman et al., 1980 |
| *Vaccinium* sp. | *Sylvilagus obscurus* | Ericaceae | C3 | Hartman and Barry, 2010 |
| *Viburnum dilatatum* | *Lepus brachyurus* | Adoxaceae | C3 | Shimizu and Shimano, 2010 |
| *Vinca minor* | *Romerolagus diazi* | Apocynaceae | C3 | Cervantes and Martinez, 1992 |
| *Viola cunninghamii* | *Lepus europaeus* | Violaceae | C3 | Flux, 1967 |
| *Viola primulifolia* | *Sylvilagus aquaticus* | Violaceae | C3 | Terrel, 1972 |
| *Vulpia muralis* | *Lepus granatensis* | Poaceae | C4 | Pauperio and Alves, 2008 |
| *Vulpia* sp. | *Oryctolagus cuniculus* | Poaceae | C4 | Alves et al., 2006 |
| *Wahlenbergia albomarginata* | *Lepus europaeus* | Campanulaceae | C3 | Flux, 1967 |
| *Weigela hortensis* | *Lepus brachyurus* | Caprifoliaceae | C3 | Shimizu and Shimano, 2010 |
| *Zanthoxylum ailanthoides* | *Pentalagus furnessi* | Rutaceae | C3 | Yamada, 2008 |
| *Zea mays* | *Lepus europaeus* | Poaceae | C4 | Reichlin et al., 2006 |

*There are C_3_, C_4_ or C_3_-C_4_ intermediates within these genera.
