# Supplementary material for: Evolutionary History of Lagomorphs in Response to Global Environmental Change
Source: PLoS One. 2013 Apr 3;8(4):e59668. doi: 10.1371/journal.pone.0059668 (PMC3616043; doi:10.1371/journal.pone.0059668)
Supplement: File S1 — Supplementary references. (DOC) [file pone.0059668.s004.doc]

**File S1.** Supplymentary references.

1. Alves J, Vingada J, Rodrigues P (2006) The wild rabbit (*Oryctolagus cuniculus* L.) diet on a sand dune area in central Portugal: a contribution towards management. Wildl Biol Prac 2: 63-70.
2. Shimizu R, Shimano K (2010) Food and habitat selection of *Lepus brachyurus* lyoni Kishida, a near-threatened species on Sado Island, Japan. Mammal Study 35: 169-177.
3. Chapman JA, Hockman JG, Ojeda C MM (1980) Sylvilagus floridanus. Mammal Spec: 1-8.
4. Hartman AC, Barry RE (2010) Survival and Winter Diet of *Sylvilagus obscurus* (Appalachian Cottontail) at Dolly Sods, West Virginia. Northeast Nat 17: 505-516.
5. Seccombe-Hett P, Turkington R (2008) Summer diet selection of snowshoe hares: a test of nutritional hypotheses. Oikos 117: 1874-1884.
6. Green JS, Flinders JT (1980) Habitat and dietary relationships of the pygmy rabbit. J Rang. Manag. 33(2): 136-142.
7. Flux JEC (1967) Hare numbers and diet in an alpine basin in New Zealand. Proc NZ Ecol Soc 14: 27-33.
8. Yamada F (2008) A review of the biology and conservation of the amami rabbit (*Pentalagus furnessi*). In: Alves PC, Ferrand N, Hacklaender K, editors. Lagomorph biology: evolution, ecology, and conservation. Berlin, Heidelberg & New York: Springer-Verlag. pp. 369-377.
9. Hudson R, Rodriguez-Martinez L, Distel H, Cordero C, Altbacker V, et al. (2005) A comparison between vegetation and diet records from the wet and dry season in the cottontail rabbit *Sylvilagus floridanus* at Ixtacuixtla, central Mexico. Acta Theriol 50: 377-389.
10. Scribner KT, Krysl LJ (1982) Summer foods of the Aububons cottontail (*Sylvilagus auduboni*: Leporidae) on Texas panhandle playa basins. Southwest Nat 27: 460-463.
11. Maccracken JG, Hansen RM (1984) Seasonal foods of blacktail jack rabbits and nuttall cottontails in southeastern Idaho. J Rang Manag 37: 256-259.
12. Pauperio J, Alves PC (2008) Diet of the Iberian hare (*Lepus granatensis*) in a mountain ecosystem. Eur J Wildl Res 54: 571-579.
13. Martin MC, Marrero P, Nogales M (2003) Seasonal variation in the diet of wild rabbits *Oryctolagus cuniculus* on a semiarid Atlantic island (Alegranza, Canarian Archipelago). Acta Theriol 48: 399-410.
14. Fa JE, Bell DJ (1990) The volcano rabbit Romerolagus diazi. In: Chapman JA, Flux JEC, editors. Rabbits, hares and pikas Status survey and conservation action plan: IUCN, Gland, Switzerland. pp. 143-146.
15. Cervantes FA, Martinez J (1992) Food habits of the rabbit *Romerolagus diazi* (Leporidae) in central Mexico. J Mammal 73: 830-834.
16. Bell DJ, Oliver WLR, Ghose RK (1990) The hispid hare *Caprolagus hispidus*; Bell DJ, Oliver WLR, Ghose RK, editors: IUCN, Gland, Switzerland. pp.128-136.
17. Reichlin T, Klansek E, Hacklaender K (2006) Diet selection by hares (*Lepus europaeus*) in arable land and its implications for habitat management. Eur J Wildl Res 52: 109-118.
18. Shipley LA, Davila TB, Thines NJ, Elias BA (2006) Nutritional requirements and diet choices of the pygmy rabbit (*Brachylagus idahoensis*): a sagebrush specialist. J Chem Ecol 32: 2455-2474.
19. Terrel TL (1972) The swamp rabbit (*Sylvilagus aquaticus*) in Indiana. American Midl Nat 87: 283-295.
20. Stewart DRM (1971) Food preferences of *Pronolagus*. Afr J Ecol 9: 163.
21. Sugimura K (1990) The Amami rabbit *Pentalagus furnessi*. In: Chapman JA, Flux JEC, editors. Rabbits, hares and pikas Status survey and conservation action plan: IUCN, Gland, Switzerland. pp. 140-142.
22. Duthie AG, Robinson TJ (1990) The African rabbits. In: Chapman JA, Flux JEC, editors. Rabbits, hares and pikas Status survey and conservation action plan: IUCN, Gland, Switzerland. pp. 121-127.
23. Lorenzo C, Carrillo-Reyes A, Gómez-Sánchez M, Velázquez A, Espinoza E (2011) Diet of the endangered Tehuantepec jackrabbit, *Lepus flavigularis*. Therya 2: 67-76.
24. Katona K, Biro Z, Szemethy L, Demes T, Nyeste M (2010) Spatial, temporal and individual variability in the autumn diet of European hare (*Lepus europaeus*) in Hungary. Acta Zool Acad Sci Hung 56: 89-101.
25. Yadav BP, Sathyakumar S, Koirala RK, Pokharel C (2008) Status, distribution and habitat use of hispid hare (*Caprolagus hispidus*) in Royal Suklaphanta Wildlife Reserve, Nepal. Tigerpaper 35: 8-14.
26. Flux JEC (1990) The Sumatran rabbit Nesolagus netscheri. In: Chapman JA, Flux JEC, editors. Rabbits, hares and pikas Status survey and conservation action plan: IUCN, Gland, Switzerland. pp. 137-139.
27. Duthie AG, Skinner JD, Robinson TJ (1989) The distribution and status of the riverine rabbit, *Bunolagus monticularis*, South Africa. Biol Conserv 47: 195-202.
28. Schmitz OJ, Hik DS, Sinclair ARE (1992) Plant chemical defense and twig selection by snowshoe hare: an optimal foraging perspective. Oikos 65: 295-300.
29. Fan NC, Jing ZC, Zhang DH (1995) Studies on the food resource niches of plateau pika and Daurian pika. Acta Theriol Sin 15: 36-40.
30. Jiang ZG, Xia WP (1985) Utilization of the food resources by plateau pika. Acta Theriol Sin 5: 251-262.
31. Millar JS, Zwickel FC (1972) Characteristics and ecological signficance of hay piles of pikas. Mammalia 36: 657-667.
32. Seccombe-Hett P, Turkington R (2008) Summer diet selection of snowshoe hares: a test of nutritional hypotheses. Oikos 117: 1874-1884.
33. Reichlin T, Klansek E, Hackländer K (2006) Diet selection by hares (*Lepus europaeus*) in arable land and its implications for habitat management. Eur J Wildl Res 52: 109-118.
34. Lorenzo C, Carrillo-Reyes A, Gómez-Sánchez M, Velázquez A, Espinoza E (2011) Diet of the endangered Tehuantepec jackrabbit, *Lepus flavigularis*. Therya 2: 67-76.
35. Martin MC, Marrero P, Nogales M (2003) Seasonal variation in the diet of wild rabbits *Oryctolagus cuniculus* on a semiarid Atlantic island (Alegranza, Canarian Archipelago). Acta Theriol 48: 399-410.
36. Hudson R, Rodriguez-Martinez L, Distel H, Cordero C, Altbacker V, et al. (2005) A comparison between vegetation and diet records from the wet and dry season in the cottontail rabbit *Sylvilagus floridanus* at Ixtacuixtla, central Mexico. Acta Theriol Sin 50: 377-389.
37. Scribner KT, Krysl LJ (1982) Summer foods of the Aububons cottontail (*Sylvilagus auduboni*: Leporidae) on Texas panhandle playa basins. Southwestern Nat 27: 460-463.
38. Zhang LB, Xu HF, Xue WJ, Jiang HR, Meng XX (2008) Winter and spring diet composition of musk deer in Feng County, Shaanxi Province. Sichuan J Zool 27: 110-114.
39. Cao YF, Su JP, Lian XM, Zhang TZ, Cui QH (2008) Food habits of Tibetan antelope (*Pantholops hodgsoni*) in the Kekexili Nature Reserve. Acta Theriol Sin 28: 14-19.
40. Hansen RM, Martin PS (1973) Ungulate Diets in the Lower Grand Canyon. J Rang Manag 26: 380-381.
41. Wang Y, Wang W (2011) Diet of Pere David's Deer (*Elaphurus davidianus*) at Milu Park in Beijing, China. Chin J Wildl 32: 65-68.
